# Psoriasis prediction from genome-wide SNP profiles

**DOI:** 10.1186/1471-5945-11-1

**Published:** 2011-01-07

**Authors:** Shenying Fang, Xiangzhong Fang, Momiao Xiong

**Affiliations:** 1 Department of Epidemiology, The University of Texas M. D. Anderson Cancer Center, Houston, Texas 77030, USA; 2 Human Genetics Center, School of Public Health, The University of Texas Health Science Center at Houston, Houston, Texas 77030, USA; 3 School of Mathematical Sciences, Peking University, Beijing 100871, P.R. China

## Abstract

**Background:**

With the availability of large-scale genome-wide association study (GWAS) data, choosing an optimal set of SNPs for disease susceptibility prediction is a challenging task. This study aimed to use single nucleotide polymorphisms (SNPs) to predict psoriasis from searching GWAS data.

**Methods:**

Totally we had 2,798 samples and 451,724 SNPs. Process for searching a set of SNPs to predict susceptibility for psoriasis consisted of two steps. The first one was to search top 1,000 SNPs with high accuracy for prediction of psoriasis from GWAS dataset. The second one was to search for an optimal SNP subset for predicting psoriasis. The sequential information bottleneck (sIB) method was compared with classical linear discriminant analysis(LDA) for classification performance.

**Results:**

The best test harmonic mean of sensitivity and specificity for predicting psoriasis by sIB was 0.674(95% CI: 0.650-0.698), while only 0.520(95% CI: 0.472-0.524) was reported for predicting disease by LDA. Our results indicate that the new classifier sIB performs better than LDA in the study.

**Conclusions:**

The fact that a small set of SNPs can predict disease status with average accuracy of 68% makes it possible to use SNP data for psoriasis prediction.

## Background

Risk classification models that utilize independent variables and outcomes through machine learning have been widely used to predict disease status in medical research. To better characterize a disease, researchers have drawn information from clinical, microarray, and single nucleotide polymorphism (SNP) data to build a disease risk model, which is then applied for clinical diagnosis and prediction of an individual's susceptibility to the disease. For example, researchers examined traditional risk factors such as age, total cholesterol, HDL cholesterol, smoking, systolic blood pressure, diabetes, and treatment for hypertension and built a classification rule with high discriminant power for diagnosing cardiovascular disease [1]. Selection of genomic biomarkers for disease classification with microarray data has been reported extensively in cancers [2-4] and other diseases[5] although in most cases high-dimensional gene expression data were obtained from a small number of observations. With the availability of high-throughput genotyping technology, data on hundreds of thousands of SNPs are available through genome-wide association studies (GWAS). One of main goals of GWAS is to identify an optimal set of SNPs that can predict disease status with greatest possible accuracy. Prediction of genetic risk will be increasingly useful in diagnosis, treatment, and prognosis of complex diseases. Exhaustive search of all possible SNP subsets to perform feature selection is computationally infeasible with a large number of features. Therefore employment of search algorithms for development of a good classifier is a challenging task.

Several feature selection methods have been used to search for an optimal SNP subset among big data sets in which feature size far exceeds the number of observations, such as search by ranked scores [6,7], or by genetic algorithm[8] or heuristics search [7,9]. Choosing SNP markers to predict disease or infer population ancestry via learning models has been reported in several studies[9-11]. For the studies performed by Brinza et al. [9] and Wang et al. [10], however, the authors used cross-validation accuracy of the optimized subset as a measure of the final performance of the classifier. This might be biased because the cross-validation accuracy for the best subset is maximized within a search algorithm wrapped around a classifier. In another genome-wide study[12], although an independent data set was preserved, the power are limited because the sizes of sample for training and test data set are insufficient. In the present study, both the training sample dataset and the independent testing sample dataset numbered more than 1000.

To evaluate the performance of a classification method on an imbalanced two-class dataset, the area under the receiver operating characteristic (ROC) curve (AUC) can be calculated. ROC plots true positive rate versus 1-true negative rate with the continuous change of threshold, and AUC can be considered as the probability that the classifier can correctly rank a randomly chosen pair of diseased and nondiseased individuals. It measures the global classification accuracy and therefore a higher AUC represents a better classifier. Lu and Elston [13] used the area under the ROC to construct a predictive genetic test on the basis of variants at several genetic loci by taking the results from some previous association researches. Cho et al.[14] applied another criterion, the harmonic mean of sensitivity and specificity (HMSS), to evaluate a classifier from an unbalanced dataset. HMSS is defined as $HMSS=2\frac{sensitivity\times specificity}{sensitivity+specificity}$, which treats sensitivity and specificity equally and is invariant to proportion of each group. This criterion was applied in the present study to assess the performance of a classifier.

In this study, a new classifier, the sequential information bottleneck (sIB), was proposed to perform classification on a big dataset with 2,798 observations and 451,724 SNPs. We first reduced the GWAS feature set into a manageable one through an effective filter method and then performed a feature selection wrapped around a classifier to identify an optimal SNP subset for predicting psoriasis. To deal with an unbalanced distribution of positive and negative observations, HMSS was used as the performance measurement of the predictive models. This study aimed to select optimal subsets of SNPs with maximum classification performance for psoriasis prediction.

## Methods

### Data sets

The dataset was obtained from the Genetic Association Information Network (GAIN) database as part of a Collaborative Association Study of psoriasis sponsored by the Foundation for the National Institutes of Health. Approval was given for the use of the data used in this study by the Genetic Association Information Network (GAIN). The data were available through dbGaP accession number phs000019.v1.p1. at URL http://dbgap.ncbi.nlm.nih.gov. All genotypes were filtered by checking for data quality [15]. A total of 1,627 subjects (941 cases and 686 controls) in the general research use (GRU) group and 1171 subjects (443 cases and 728 controls) in an autoimmune disease only (ADO) group were finally used in this study. Each case of psoriasis was diagnosed by a dermatologist. Controls were at least 18 years old and had no cognate relative with a known diagnosis of psoriasis. Both cases and controls agreed to sign in the consent contract. DNA for each of the participants was genotyped with the Perlegen 500 K array. It was assumed that high-dimension data mining tools, together with feature selection methods, might provide clues for locating the genes for predicting the disease on a genome-wide scale.

### Experiments

Each genotype was assigned a value of 0, 1 or 2, where 0 and 2 denoted homozygotes of minor and majror alleles, respectively and 1 represented heterozygotes. Missing data were replaced with the majority category for each SNP feature. We trained the model based on the GRU dataset and tested it through ADO dataset. Within the training dataset, the 1,000 most predictive SNPs were chosen through one criterion to reduce the space dimension to a manageable size. The training set was further split into five non-overlapping subsets of equal size. Four of these subsets were used for training, and the remaining one for evaluation. The wrapper approach follows the feature subset selection algorithm "wrapped around" the mining algorithms, such as sIB or linear discriminant analysis (LDA). Two search algorithms, forward selection (FS) and sequential forward floating selection (SFFS), were compared. The model with best cross-validation evaluation was chosen and tested on the independent test set for each learning algorithm. The results for the test set represent an estimate of generalization accuracy. This accuracy was plotted over the number of variables included in the model so that the optimal number of variables could be determined. For each promising subset of markers, we further ran 200 bootstrap iterations to estimate 95% confidence interval for the accuracy.

LDA is a classifier that separates the two classes by determining the projection matrix that maximizes the ratio of between-class covariance to within-class covariance. LDA is simple, fast and often produces models with accuracy comparable to more complex mthods. sIB is a new, unsupervised method that can be adopted for classification tasks. Given a joint distribution *p*(*X;Y*), the principle of this method is to find the cluster variable C that compresses the original information X while trying to preserve the relevant features in X with respect to information about Y. By introducing a Lagrange multiplier, β, the information bottleneck function is constructed as *L*[*P*(*c *| *x*)] = *I*(*C*; *X*) - *β I *(*C*; *Y*), where *I*(*C*; *X*) represents mutual information between C and X and *I*(*C*; *Y*) means mutual information between C and Y. By minimizing this function, we can solve for *P*(*c *| *x*), based on which x is mapped into c. In the sIB algorithm, this conditional probability is deterministic, which means that x is assigned to a cluster with probability 1 and all other clusters with probability 0. By randomly initializing the partitions several times, the clustering that minimizes the loss function *L *is finally chosen. After clusters are built, each one is labelled by its dominant category [16]. sIB was demonstrated to have a better performance than all other unsupervised classification methods(agglomerative, K-means and other sequential procedures) by a significant gap and was comparable to the naïve Bayes method [17].

All programming analysis was done with MATLAB student version 7.1 and the SAS system (Cary, NC).

## Results

To reduce the feature subsets to a manageable size, we first used training HMSS of LDA as a filter criterion to obtain the candidate 1,000 SNPs for the second-stage marker selection. The psoriasis data set consists of two independent studies. The first study (cases: 941 and controls: 686) was used as a training data set and the second study (cases: 443 and controls: 728) was used as a test data set. We used LDA with HMSS as a selection criterion and selected best 1000 SNPs from 451,724 SNPs genotyped in the training data set (Additional file 1, Table S1).

**Figure 1:**
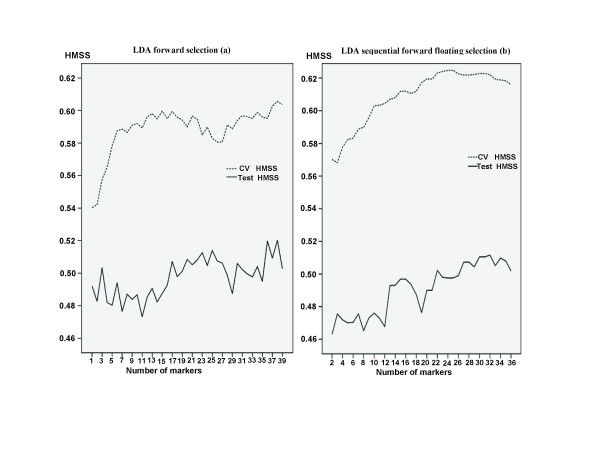
**Cross-validation HMSS (CVhmss) and test HMSS (thmss) by the number of markers**. The markers were identified for predicting psoriasis using LDA classifier with forward selection(a) and sequential forward floating selection (b) algorithms, respectively.

To identify an optimal subset of predictive SNPs using a LDA classifier, the study consists of two steps. In the first step, we split the training set into five non-overlapping subsets of equal size. Four of them were used for training, and the remaining one for cross-validation. We used LDA as a classifier and HMSS evaluated in the cross-validation data set as a criterion to select an optimal subset of SNPs for prediction by FS or SFFS algorithms (see Methods). In the second step, we used the test data set to calculate HMSS of the optimal subset of SNPs selected from the first step. To determine how many SNPs should be used for prediction, we plotted Figure 1 showing cross-validation HMSS and test HMSS versus the number of markers searched through two feature selection algorithms. Inclusion of more SNP markers in FS did not lead to a significant change of test HMSS(Figure 1(a)). The best subset consisted of 38 SNPs and its test HMSS was 0.520(95% CI: 0.472-0.524). Because this method easily generates nested subsets of SNPs and optimal large subsets might not include optimal subsets with small sizes, FS does not give an optimal result. We also used SFFS for feature selection(Figure 1(b), by backtracking after each inclusion, SFFS started from a 2-SNP subset and all possible 2-SNP combinations were evaluated. Although cross-validation HMSS for SFFS was in general higher than that for FS, the test HMSS for SFFS was, in general, lower than that for FS. The highest value for test HMSS in SFFS was 0.512(95% CI: 0.472-0.523), produced by an optimal subset of 32 SNPs.

**Figure 2:**
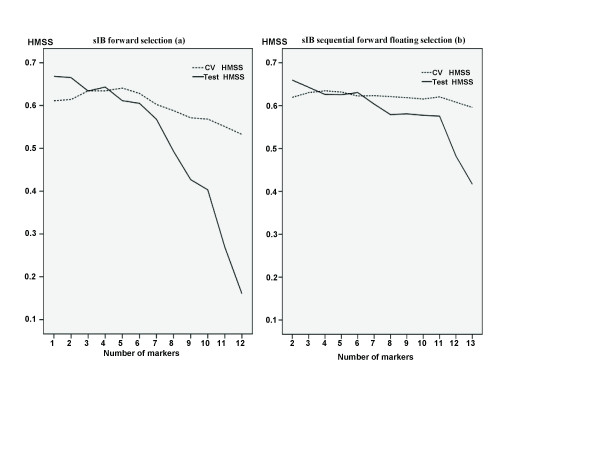
**Cross-validation HMSS(CVhmss) and test HMSS(thmss) by the number of markers**. The markers were determined for predicting psoriasis status that can be obtained by sIB classifier with forward selection(a) and sequential forward floating selection (b), respectively.

To find a subset of SNPs with maximum predictive ability, two different feature selection algorithms wrapped around the sIB classifier were performed. Cross-validation HMSS and test HMSS were plotted by the number of markers searched through each feature selection method (Figure 2). In the process of FS(Figure 2(a)), although cross-validation HMSS reached its top value when the size of subsets reached six, the test HMSS for FS algorithm reached its highest value 0.668(95% CI: 0.641-0.694) with only one SNP marker and decreased with additional markers. SFFS(Figure 2(b)) started from an optimal 2-SNP subset obtained by evaluating all combinations of 2 SNPs. Similar to FS, test HMSS for SFFS reached its highest value 0.659(95% CI: 0.633-0.683) with a subset of 2 SNPs and decreased with additional markers.

Table 1 and Additional file 1, Table S2 list all optimal SNP subsets for each search algorithm using the LDA or sIB classifier. For the LDA method, although the subset of SNPs rs10958357 and rs7973936 had the highest test HMSS of 0.556(95% CI: 0.471-0.525), it was picked post hoc and spuriously inflated with the test HMSS not covered by 95% CI, therefore, we used mean value of Bootstrap sampling--0.498 as the point value estimate for the test HMSS. The reliable test HMSS was given by the subset of 38 SNPs from forward selection. Its test HMSS reached 0.520(95% CI: 0.472-0.524), and its cross-validation HMSS was 0.500(95% CI: 0.478-0.520). For the sIB method, the best subset of SNPs is rs12191877 and rs4953658 with test HMSS reaching 0.674(95% CI: 0.650-0.698) and cross-validation HMSS 0.557(95% CI: 0.014-0.633). In general, sIB performed better than LDA for all search algorithms, and point estimate results for sIB were all located within 95% confidence interval

**Table 1:** Optimal SNP subsets using LDA or sIB for predicting psoriasis with average accuracy and 95% confidence interval estimated from Bootstrap re-sampling

Subsets	Components (dbSNP_rs on chromosome)	CV HMSS (Bootstrap mean and 95% CI)	Total CV accuracy (Bootstrap mean and 95% CI)	Test HMSS (Bootstrap mean and 95% CI)	Total test accuracy (Bootstrap mean and 95% CI)
**LDA**					

1 SNP*	rs10905106 on 10	0.498(0.498, 0.475-0.518)	0.495(0.496, 0.474-0.516)	0.544(0.494, 0.469-0.519)	0.553(0.508, 0.486-0.532)

2 SNPs*	rs10958357 on 8 rs7973936 on 12	0.486(0.499, 0.480-0.520)	0.495(0.500, 0.481-0.521)	0.556(0.498, 0.471-0.525)	0.565(0.512, 0.491-0.535)

1 SNP^∆^	rs4375421 on 11	0.540(0.497, 0.474-0.519)	0.540(0.498, 0.476-0.518)	0.492(0.500, 0.476-0.529)	0.491(0.499, 0.474-0.529)

2 SNPs^∆^	rs950753 on 3 rs7058025 on X	0.570(0.493, 0.468-0.514)	0.575(0.508, 0.486-0.530)	0.463(0.476, 0.451-0.502)	0.459(0.473, 0.450-0.498)

FS	38 SNPs	0.604(0.500, 0.478-0.520)	0.622(0.503, 0.482-0.523)	**0.520(0.496, 0.472-0.524)**	**0.514(0.491, 0.466-0.518)**

SFFS	32 SNPs	0.622(0.497, 0.475-0.520)	0.622(0.502, 0.479-0.525)	0.512(0.498, 0.472-0.523)	0.509(0.498, 0.473-0.522)

**sIB**					

1 SNP*	rs12191877 on 6	0.611(0.605, 0.563-0.630)	0.611(0.608, 0.580-0.631)	0.668(0.668, 0.641-0.694)	0.699(0.698, 0.676-0.720)

2 SNPs*	rs12191877 on 6 rs4953658 on 2	0.557(0.444, 0.014-0.633)	0.574(0.550, 0.426-0.633)	**0.674(0.674, 0.650-0.698)**	**0.685(0.684, 0.662-0.707)**

FS	rs12191877 on 6	0.611(0.605, 0.563-0.630)	0.611(0.608, 0.580-0.631)	0.668(0.668, 0.641-0.694)	0.699(0.698, 0.676-0.720)

SFFS	rs2844627 on 6 rs7773175 on 6	0.619(0.617, 0.576-0.641)	0.616(0.615, 0.585-0.638)	0.659(0.658, 0.633-0.683)	0.677(0.676, 0.655-0.699)

* The best test HMSS among all subsets

^∆ ^Test HMSS for the subset with the best CV HMSS

HMSS as a criterion gives equal weight to both sensitivity and specificity. But in practice, there have also been concerns regarding prediction accuracy in one group given that the accuracy in the other group is reasonable. In this study, for example, to determine how much accuracy a SNP subset can reach to predict a normal status if its prediction accuracy among cases is at least 0.4, we observed the top 20 ranked SNP subsets based on cross-validation accuracies in controls for all SNPs with accuracies not less than 0.4 among cases. Table 2 presents the highest test accuracies for predicting non-psoriasis among controls with test accuracy equal to or greater than 0.4 among cases, or for predicting psoriasis among cases with test accuracy equal to or greater than 0.4 among controls, determined by using a LDA or sIB classifier through 1 or 2 SNPs. Generally speaking, sIB yielded higher test accuracy than LDA. If the test accuracy among cases was required to be at least 0.4, then the best test accuracies among controls were 0.582(95% CI: 0.555-0.613) by LDA through rs7507133 and 0.850 (95% CI: 0.826-0.870) by sIB through a combination of rs12191877 and rs3823418. On the other hand, if the test accuracy among controls was required to be at least 0.4, then the best test accuracies among cases were 0.607(95% CI: 0.558-0.628) by LDA through rs231390 and 0.749(95% CI: 0.718-0.779) by sIB through rs1265078 and rs1466215.

**Table 2:** 1 SNP or 2-SNP subsets with the highest group accuracies using LDA and sIB for predicting psoriasis

Subsets	Components	Test accuracy among controls with ≥0.4 among cases(Bootstrap mean and 95% CI)	Components	Test accuracy among cases with ≥0.4 among controls(Bootstrap mean and 95% CI)
**LDA**				

1 SNP	rs7507133 on 19	**0.582(0.586, 0.555-0.613)**	rs231390 on 2	**0.607(0.591, 0.558-0.628)**
2 SNPs,based on all combinations	___*	___*	____ ^Δ^	____ ^Δ^
**sIB**				
1 SNP	rs12191877 on 6	0.761(0.760, 0.735-0.785)	rs7773175 on 6	0.731(0.731, 0.694-0.764)
2 SNPs-based on all combinations	rs12191877 on 6 rs3823418 on 6	**0.850(0.850, 0.826-0.870)**	rs1265078 on 6 rs1466215 on 4	**0.749(0.748, 0.718-0.779)**

* No subsets with test accuracies ≥0.4 among cases

ΔNo subsets with test accuracies ≥0.4 among controls

Table 3 and Additional file 1, Table S3 list 20 SNPs with the best training HMSS for classifying psoriasis: 15 have *P*-values smaller than the cut-off value 1.11 × 10^-7 ^by a chi-square test. This demonstrates that our classification methods are consistent with chi-square test in terms of detecting valuable SNP markers.

**Table 3:** Classification accuracy and chi-square test for 20 SNPs with the highest training HMSS by LDA for predicting psoriasis

	Chr	LDA		sIB			
				
SNP_RS		Training HMSS	Test HMSS	Training HMSS	Test HMSS	*P*-value(GRU) *	*P*-value(ADO) *
rs12191877	6	0.611	0.315	0.611	0.668	**0**	**0**
rs2894207	6	0.603	0.387	0.603	0.657	**0**	**1.11 × 10**^**-16**^
rs3130517	6	0.600	0.414	0.600	0.608	**0**	**4.33 × 10**^**-15**^
rs2394895	6	0.598	0.425	0.598	0.620	**0**	**1.13 × 10**^**-14**^
rs2844627	6	0.598	0.413	0.598	0.627	**0**	**0**
rs3130713	6	0.597	0.400	0.597	0.599	**5.55 × 10**^**-16**^	**1.52 × 10**^**-14**^
rs3130467	6	0.596	0.415	0.596	0.605	**1.11 × 10**^**-16**^	**2.73 × 10**^**-14**^
rs9468933	6	0.595	0.321	0.595	0.656	**0**	**0**
rs7773175	6	0.585	0.416	0.585	0.604	**0**	**1.33 × 10**^**-15**^
rs6861600	5	0.569	0.456	0.569	0.544	7.48 × 10^-4^	**2.32 × 10**^**-8**^
rs9380237	6	0.569	0.405	0.569	0.628	**0**	**1.38 × 10**^**-10**^
rs6887695	5	0.568	0.454	0.568	0.546	7.46 × 10^-4^	**3.96 × 10**^**-8**^
rs3823418	6	0.568	0.342	0.120	0.142	**0**	**1.11 × 10**^**-16**^
rs1265078	6	0.565	0.434	0.565	0.561	**5.29 × 10**^**-11**^	**3.44 × 10**^**-12**^
rs2647087	6	0.564	0.443	0.564	0.573	**1.25 × 10**^**-8**^	**1.21 × 10**^**-8**^
rs2858333	6	0.564	0.444	0.564	0.572	**5.65 × 10**^**-8**^	**1.23 × 10**^**-8**^
rs3132965	6	0.564	0.434	0.564	0.574	**2.57 × 10**^**-9**^	**6.62 × 10**^**-9**^
rs10947208	6	0.563	0.442	0.563	0.554	2.77 × 10^-5^	**3.71 × 10**^**-8**^
rs9266846	6	0.562	0.458	0.562	0.548	1.31 × 10^-5^	3.03 × 10^-7^
rs497150	22	0.562	0.486	0.562	0.490	0.22062	1.67 × 10^-5^

* cut-off *P*-value = 1.11 × 10^-7 ^(0.05/451724)

## Discussion

The purpose of this study is to address the feasibility of using SNPs for diagnosis of psoriasis. To achieve this, we addressed two issues. The first issue is what classification method should be used. We propose to use a new adaptive classification method, sIB wrapped inside feature selection methods, to predict disease occurrence. By first extracting a fixed number of clusters that minimizes the loss of mutual information between the input features and the outcome variable, and then assigning the class label to each cluster by a majority rule, this method has been proved to perform better than linear discriminant analysis in this study.

The second issue is how to select a subset of SNPs from half millions of SNPs. Accessibility of millions of DNA variations makes it possible to use SNPs for predicting common diseases. Because the number of combinations of SNPs is extremely large, it is necessary to select a subset of informative SNP markers that can accurately predict the disease. In this study, we first selected the 1,000 most predictive SNPs through an effective filter method and then used a feature selection algorithm wrapped around a classifier to identify an optimal SNP subset for predicting the disease. In general, searching for an optimal subset through cross-validation criterion gives an overfitting estimate of subset's performance and makes poor generalization. It is strange that inclusion of more SNPs in the optimal sets of SNPs with 1 or 2 SNPs through an exhaustive search does not always improve accuracy. sIB can be adopted to predict the outcome, and in our study its ability to predict the disease status was better than that of traditional LDA. The best test HMSS for predicting psoriasis using a subset of SNPs with rs12191877 and rs4953658 through sIB was 0.674. In terms of group prediction accuracy for the study of psoriasis, if accuracy in the test samples among cases was required to be not less than 0.4, the highest accuracy of sIB among controls reached 0.850 by a combination of rs12191877 and rs3823418. On the other hand, if prediction precision among control test samples was required to be at least 0.4, the best accuracy of sIB among case test samples reached 0.749 by a combination of SNPs rs1265078 and rs1466215.

An initial filter approach is fast and simple to perform. The reason that features were first filtered through LDA HMSS rather than through LDA classification accuracy is that LDA HMSS can be applicable in imbalanced data without resampling the original dataset. Since filter approaches look at each feature independently and the best subset may not consist of the best features selected individually[18], selecting the top ranked features might not generate an optimal subset. They can, however, work as a preliminary technique to obtain a candidate subset with a manageable size. More intensive feature selection and model learning methods should be performed in the next step. In this study, the most predictive 1000 HMSS SNPs were selected as a filter subset and the second-stage wrapper approach was based on this subset.

In this study, using a variable ranking method to discard the lowest scoring SNPs through a filter method could potentially lose some variables that prove to be important in a wrapper process. Guyon and Elisseeff showed that a feature that is useless by itself (least separation of the target variable) can improve other features' classification performances if they are combined together[19]. Since exhaustive search for all possible combinations of SNPs for best prediciton precision is computationally infeasible, using top 1,000 SNPs with the highest individual classification accuracy may miss the optimal sets of SNPs with the highest classification accuracy. Although this study shows that we can predict the risk of psoriasis with an identified subset of SNP markers, this doesn't mean that those markers are functionally associated with the feature of interest. The identified SNPs in this study mainly serve as prediction variables.

## Conclusions

We propose a new adaptive classification method, sIB wrapped inside feature selection methods, to predict disease occurrence. By first extracting a fixed number of clusters that minimizes the loss of mutual information between the input features and the outcome variable, and then assigning the class label to each cluster by a majority rule, this method has been proved to perform better than linear discriminant analysis in this dataset. Searching for an optimal subset through cross-validation criterion gives an overfitting estimate of subset's test performance. Starting from an exhaustive search of a small subset, incorporation of more SNPs through a heuristic algorithm does not always improve accuracy. Although SFFS can be applied to prevent from the nesting effect of FS, it can lead to overfitting because it involves more complex subset states and thus does not outperform FS.

Although our classification methods achieved high prediction accuracy in this study, determining the statistical significance of those models requires more cost-effective methods or efficient computing system, neither of which is available currently in our genome-wide study. We should also note that the purpose of this study was to identify subsets of SNPs with high predictive ability, and that SNPs with good discriminant power are not necessarily causal markers for the disease. The fact that a small set of SNPs can predict disease status with average accuracy of 68% makes it possible to use SNP data for psoriasis prediction.

## Competing interests

The authors declare that they have no competing interests.

## Authors' contributions

SF carried out the genetic studies, performed data analysis and drafted the manuscript. XF participated in the design of the study and performed the statistical analysis. MX conceived of the study, and participated in its design and coordination and helped to draft the manuscript. All authors read and approved the final manuscript.

## Pre-publication history

The pre-publication history for this paper can be accessed here:

http://www.biomedcentral.com/1471-5945/11/1/prepub

## Supplementary Material

Additional file 1 **Supplemental table S1-S3**. Table S1. Selected 1000 SNPs based on HMSS in the training general research use(GRU) group. Table S2. Prediction accuracy with Bootstrap mean and 95% confidence interval for optimal SNP subsets using LDA or sIB for predicting psoriasis. Table S3. Classification accuracy (Bootstrap mean and 95% CI) and chi-square test for 20 SNPs with the highest training HMSS by LDA for predicting psoriasis Click here for file
