# Supplementary material for: Psoriasis prediction from genome-wide SNP profiles
Source: BMC Dermatol. 2011 Jan 7;11:1. doi: 10.1186/1471-5945-11-1 (PMC3022824; doi:10.1186/1471-5945-11-1)
Supplement: Additional file 1 — Supplemental table S1-S3. Table S1. Selected 1000 SNPs based on HMSS in the training general research use(GRU) group. Table S2. Prediction accuracy with Bootstrap mean and 95% confidence interval for optimal SNP subsets using LDA or sIB for predicting psoriasis. Table S3. Classification accuracy (Bootstrap mean and 95% CI) and chi-square test for 20 SNPs with the highest training HMSS by LDA for predicting psoriasis [file 1471-5945-11-1-S1.DOC]

Table S1. Selected 1000 SNPs based on HMSS in the training general research use(GRU) group

| chromosome | dbSNP_RS | Training HMSS | Training accuracy among controls | Training accuracy among cases | Total training accuracy |
| --- | --- | --- | --- | --- | --- |
| 6 | rs12191877 | 0.6113 | 0.7216 | 0.5303 | 0.6109 |
| 6 | rs2894207 | 0.6033 | 0.6283 | 0.5802 | 0.6005 |
| 6 | rs3130517 | 0.6001 | 0.551 | 0.6589 | 0.6134 |
| 6 | rs2394895 | 0.5981 | 0.5948 | 0.6015 | 0.5986 |
| 6 | rs2844627 | 0.5975 | 0.5437 | 0.6631 | 0.6128 |
| 6 | rs3130713 | 0.5966 | 0.551 | 0.6504 | 0.6085 |
| 6 | rs3130467 | 0.5962 | 0.5423 | 0.6621 | 0.6116 |
| 6 | rs9468933 | 0.5948 | 0.7216 | 0.5058 | 0.5968 |
| 6 | rs7773175 | 0.5853 | 0.5131 | 0.6812 | 0.6103 |
| 5 | rs6861600 | 0.5693 | 0.57 | 0.5685 | 0.5691 |
| 6 | rs9380237 | 0.5688 | 0.4985 | 0.6621 | 0.5931 |
| 5 | rs6887695 | 0.5678 | 0.5671 | 0.5685 | 0.5679 |
| 6 | rs3823418 | 0.5677 | 0.7172 | 0.4697 | 0.5741 |
| 6 | rs1265078 | 0.5653 | 0.4811 | 0.6854 | 0.5993 |
| 6 | rs2647087 | 0.5642 | 0.5248 | 0.61 | 0.5741 |
| 6 | rs2858333 | 0.5641 | 0.5262 | 0.6079 | 0.5734 |
| 6 | rs3132965 | 0.5641 | 0.6341 | 0.508 | 0.5612 |
| 6 | rs10947208 | 0.5625 | 0.5845 | 0.542 | 0.5599 |
| 6 | rs9266846 | 0.562 | 0.5598 | 0.5643 | 0.5624 |
| 22 | rs497150 | 0.5616 | 0.6108 | 0.5197 | 0.5581 |
| 6 | rs13437088 | 0.5609 | 0.5525 | 0.5696 | 0.5624 |
| 6 | rs1052248 | 0.5601 | 0.5656 | 0.5547 | 0.5593 |
| 6 | rs3130048 | 0.5601 | 0.5612 | 0.559 | 0.5599 |
| 6 | rs9266825 | 0.5588 | 0.5554 | 0.5622 | 0.5593 |
| 6 | rs7756521 | 0.5585 | 0.6589 | 0.4846 | 0.5581 |
| 6 | rs9295991 | 0.5581 | 0.5481 | 0.5685 | 0.5599 |
| 6 | rs9266845 | 0.5576 | 0.5481 | 0.5675 | 0.5593 |
| 22 | rs2139278 | 0.5572 | 0.602 | 0.5186 | 0.5538 |
| 6 | rs176095 | 0.5547 | 0.6064 | 0.5112 | 0.5513 |
| 6 | rs1051792 | 0.5546 | 0.516 | 0.5994 | 0.5642 |
| 6 | rs8365 | 0.5541 | 0.6822 | 0.4665 | 0.5575 |
| 6 | rs4711229 | 0.5513 | 0.4869 | 0.6355 | 0.5728 |
| 17 | rs11656155 | 0.5503 | 0.5612 | 0.5399 | 0.5489 |
| 6 | rs9266813 | 0.5502 | 0.5131 | 0.593 | 0.5593 |
| 6 | rs162297 | 0.5499 | 0.5627 | 0.5377 | 0.5482 |
| 17 | rs12451030 | 0.5498 | 0.5671 | 0.5335 | 0.5476 |
| 6 | rs916570 | 0.5496 | 0.5729 | 0.5282 | 0.547 |
| 6 | rs162295 | 0.5491 | 0.5656 | 0.5335 | 0.547 |
| 6 | rs240993 | 0.549 | 0.5496 | 0.5484 | 0.5489 |
| 6 | rs9359208 | 0.549 | 0.5175 | 0.5845 | 0.5562 |
| 5 | rs10866712 | 0.5489 | 0.4869 | 0.6291 | 0.5691 |
| 6 | rs2021729 | 0.5487 | 0.5 | 0.6079 | 0.5624 |
| 11 | rs7925479 | 0.5486 | 0.5743 | 0.525 | 0.5458 |
| 6 | rs1611350 | 0.5482 | 0.5569 | 0.5399 | 0.547 |
| 11 | rs947877 | 0.5482 | 0.5685 | 0.5292 | 0.5458 |
| 6 | rs9380240 | 0.548 | 0.6166 | 0.4931 | 0.5452 |
| 1 | rs2201841 | 0.5476 | 0.5219 | 0.576 | 0.5532 |
| 9 | rs2988071 | 0.5474 | 0.5656 | 0.5303 | 0.5452 |
| 9 | rs7041937 | 0.5472 | 0.5292 | 0.5664 | 0.5507 |
| 17 | rs12601221 | 0.5471 | 0.5219 | 0.5749 | 0.5526 |
| 6 | rs1590560 | 0.5469 | 0.5277 | 0.5675 | 0.5507 |
| 11 | rs12146673 | 0.5467 | 0.535 | 0.559 | 0.5489 |
| 6 | rs16899207 | 0.5467 | 0.5379 | 0.5558 | 0.5482 |
| 6 | rs16899213 | 0.5464 | 0.5466 | 0.5462 | 0.5464 |
| 6 | rs241453 | 0.5464 | 0.5394 | 0.5537 | 0.5476 |
| 9 | rs3002339 | 0.5461 | 0.5641 | 0.5292 | 0.5439 |
| 6 | rs3094205 | 0.5461 | 0.4475 | 0.7003 | 0.5937 |
| 6 | rs1233487 | 0.5461 | 0.5977 | 0.5027 | 0.5427 |
| 6 | rs1886985 | 0.5458 | 0.5248 | 0.5685 | 0.5501 |
| 1 | rs2133173 | 0.5455 | 0.5525 | 0.5388 | 0.5446 |
| 2 | rs13026755 | 0.5453 | 0.6035 | 0.4973 | 0.5421 |
| 21 | rs2836824 | 0.5452 | 0.5321 | 0.559 | 0.5476 |
| 6 | rs9468935 | 0.545 | 0.519 | 0.5739 | 0.5507 |
| 6 | rs2844535 | 0.5447 | 0.5685 | 0.5228 | 0.5421 |
| 12 | rs7973612 | 0.5446 | 0.6064 | 0.4942 | 0.5415 |
| 6 | rs3757340 | 0.5445 | 0.5233 | 0.5675 | 0.5489 |
| 15 | rs3829480 | 0.5445 | 0.6079 | 0.4931 | 0.5415 |
| 6 | rs9461688 | 0.5444 | 0.5204 | 0.5707 | 0.5495 |
| 4 | rs6854827 | 0.5444 | 0.5204 | 0.5707 | 0.5495 |
| 2 | rs12471315 | 0.5442 | 0.5554 | 0.5335 | 0.5427 |
| 6 | rs12192713 | 0.5441 | 0.5685 | 0.5218 | 0.5415 |
| 1 | rs664533 | 0.5441 | 0.5671 | 0.5228 | 0.5415 |
| 3 | rs16859665 | 0.5439 | 0.5481 | 0.5399 | 0.5433 |
| 6 | rs1419675 | 0.5439 | 0.5758 | 0.5154 | 0.5409 |
| 1 | rs1542875 | 0.5439 | 0.5277 | 0.5611 | 0.547 |
| 1 | rs1542875 | 0.5439 | 0.5277 | 0.5611 | 0.547 |
| 6 | rs2294780 | 0.5438 | 0.5248 | 0.5643 | 0.5476 |
| 4 | rs1250126 | 0.5437 | 0.5423 | 0.5452 | 0.5439 |
| 17 | rs8081289 | 0.5437 | 0.5554 | 0.5324 | 0.5421 |
| 17 | rs17708843 | 0.5436 | 0.5933 | 0.5016 | 0.5403 |
| 2 | rs1080207 | 0.5435 | 0.5977 | 0.4984 | 0.5403 |
| 6 | rs13195441 | 0.5434 | 0.5437 | 0.543 | 0.5433 |
| 22 | rs479229 | 0.5434 | 0.6035 | 0.4942 | 0.5403 |
| 1 | rs12138303 | 0.5434 | 0.5525 | 0.5345 | 0.5421 |
| 6 | rs3130573 | 0.5433 | 0.4504 | 0.6844 | 0.5857 |
| 3 | rs10935878 | 0.5431 | 0.5262 | 0.5611 | 0.5464 |
| 16 | rs16952347 | 0.543 | 0.5233 | 0.5643 | 0.547 |
| 1 | rs1724956 | 0.543 | 0.5496 | 0.5367 | 0.5421 |
| 6 | rs1265048 | 0.543 | 0.5933 | 0.5005 | 0.5396 |
| 16 | rs1477096 | 0.5429 | 0.516 | 0.5728 | 0.5489 |
| 6 | rs4946640 | 0.5429 | 0.5816 | 0.509 | 0.5396 |
| 6 | rs3906276 | 0.5429 | 0.5306 | 0.5558 | 0.5452 |
| 15 | rs524908 | 0.5429 | 0.5277 | 0.559 | 0.5458 |
| 4 | rs17617811 | 0.5429 | 0.6137 | 0.4867 | 0.5403 |
| 10 | rs12781461 | 0.5426 | 0.5729 | 0.5154 | 0.5396 |
| 18 | rs1918674 | 0.5426 | 0.535 | 0.5505 | 0.5439 |
| 6 | rs3094672 | 0.5423 | 0.5816 | 0.508 | 0.539 |
| 6 | rs3131636 | 0.5423 | 0.5802 | 0.509 | 0.539 |
| 6 | rs742697 | 0.5422 | 0.5466 | 0.5377 | 0.5415 |
| 5 | rs10475933 | 0.5422 | 0.5466 | 0.5377 | 0.5415 |
| 18 | rs602422 | 0.5421 | 0.535 | 0.5494 | 0.5433 |
| X | rs5929597 | 0.5421 | 0.535 | 0.5494 | 0.5433 |
| 13 | rs9301999 | 0.542 | 0.5627 | 0.5228 | 0.5396 |
| X | rs1205782 | 0.5419 | 0.5612 | 0.5239 | 0.5396 |
| 16 | rs2059297 | 0.5419 | 0.5335 | 0.5505 | 0.5433 |
| 6 | rs2647012 | 0.5418 | 0.6399 | 0.4697 | 0.5415 |
| 6 | rs4140545 | 0.5418 | 0.586 | 0.5037 | 0.5384 |
| 5 | rs13163870 | 0.5417 | 0.4942 | 0.5994 | 0.555 |
| 6 | rs3130043 | 0.5417 | 0.5962 | 0.4963 | 0.5384 |
| 5 | rs9885032 | 0.5417 | 0.5962 | 0.4963 | 0.5384 |
| 6 | rs362522 | 0.5417 | 0.4548 | 0.6695 | 0.579 |
| 6 | rs10947126 | 0.5417 | 0.6122 | 0.4857 | 0.539 |
| 16 | rs7195745 | 0.5416 | 0.5977 | 0.4952 | 0.5384 |
| 10 | rs2588948 | 0.5416 | 0.5015 | 0.5887 | 0.5519 |
| 5 | rs2227284 | 0.5415 | 0.5044 | 0.5845 | 0.5507 |
| 6 | rs2670123 | 0.5414 | 0.5058 | 0.5824 | 0.5501 |
| 8 | rs4543587 | 0.5414 | 0.5714 | 0.5143 | 0.5384 |
| 9 | rs12555766 | 0.5413 | 0.57 | 0.5154 | 0.5384 |
| 3 | rs9784333 | 0.5413 | 0.4942 | 0.5983 | 0.5544 |
| 13 | rs12865306 | 0.5413 | 0.5219 | 0.5622 | 0.5452 |
| 6 | rs9365285 | 0.5412 | 0.5685 | 0.5165 | 0.5384 |
| 3 | rs234009 | 0.5412 | 0.5481 | 0.5345 | 0.5403 |
| 9 | rs10812321 | 0.5412 | 0.5481 | 0.5345 | 0.5403 |
| 16 | rs8057543 | 0.5411 | 0.586 | 0.5027 | 0.5378 |
| 10 | rs12412014 | 0.5411 | 0.5845 | 0.5037 | 0.5378 |
| 6 | rs2844724 | 0.5411 | 0.5816 | 0.5058 | 0.5378 |
| 19 | rs628258 | 0.5411 | 0.5933 | 0.4973 | 0.5378 |
| 6 | rs10947125 | 0.5411 | 0.6108 | 0.4857 | 0.5384 |
| 18 | rs7234917 | 0.5411 | 0.6341 | 0.4718 | 0.5403 |
| 3 | rs17049438 | 0.5411 | 0.5466 | 0.5356 | 0.5403 |
| 6 | rs7194 | 0.5411 | 0.6399 | 0.4687 | 0.5409 |
| 2 | rs10191126 | 0.541 | 0.5787 | 0.508 | 0.5378 |
| 2 | rs17014938 | 0.541 | 0.516 | 0.5685 | 0.5464 |
| X | rs7065033 | 0.5409 | 0.5306 | 0.5515 | 0.5427 |
| 18 | rs665445 | 0.5409 | 0.5408 | 0.5409 | 0.5409 |
| 6 | rs2534792 | 0.5408 | 0.4898 | 0.6036 | 0.5556 |
| 6 | rs2844571 | 0.5407 | 0.5087 | 0.577 | 0.5482 |
| 2 | rs10496065 | 0.5407 | 0.519 | 0.5643 | 0.5452 |
| 17 | rs632078 | 0.5406 | 0.5102 | 0.5749 | 0.5476 |
| 1 | rs11585207 | 0.5405 | 0.5466 | 0.5345 | 0.5396 |
| 6 | rs2844573 | 0.5405 | 0.5117 | 0.5728 | 0.547 |
| 6 | rs6927461 | 0.5405 | 0.5204 | 0.5622 | 0.5446 |
| 14 | rs11157138 | 0.5405 | 0.551 | 0.5303 | 0.539 |
| 6 | rs9357152 | 0.5404 | 0.5641 | 0.5186 | 0.5378 |
| 11 | rs2060477 | 0.5404 | 0.5277 | 0.5537 | 0.5427 |
| 2 | rs3094416 | 0.5404 | 0.5175 | 0.5654 | 0.5452 |
| 6 | rs1077394 | 0.5404 | 0.5758 | 0.509 | 0.5372 |
| 15 | rs891343 | 0.5404 | 0.5306 | 0.5505 | 0.5421 |
| 7 | rs4724413 | 0.5403 | 0.5335 | 0.5473 | 0.5415 |
| 3 | rs9823664 | 0.5403 | 0.5554 | 0.526 | 0.5384 |
| 2 | rs17030410 | 0.5403 | 0.5219 | 0.56 | 0.5439 |
| 13 | rs4132816 | 0.5403 | 0.5729 | 0.5112 | 0.5372 |
| 17 | rs12943490 | 0.5402 | 0.5364 | 0.5441 | 0.5409 |
| 6 | rs6936689 | 0.5401 | 0.5481 | 0.5324 | 0.539 |
| 6 | rs2524222 | 0.5401 | 0.6152 | 0.4814 | 0.5378 |
| 4 | rs11730661 | 0.5401 | 0.5598 | 0.5218 | 0.5378 |
| 6 | rs6916769 | 0.5401 | 0.5233 | 0.5579 | 0.5433 |
| 11 | rs503676 | 0.5401 | 0.516 | 0.5664 | 0.5452 |
| 18 | rs2456 | 0.54 | 0.5204 | 0.5611 | 0.5439 |
| 5 | rs10052016 | 0.54 | 0.4927 | 0.5972 | 0.5532 |
| 2 | rs2356470 | 0.54 | 0.4913 | 0.5994 | 0.5538 |
| 16 | rs4389136 | 0.5399 | 0.5379 | 0.542 | 0.5403 |
| 6 | rs17465706 | 0.5399 | 0.4898 | 0.6015 | 0.5544 |
| 1 | rs6698102 | 0.5399 | 0.551 | 0.5292 | 0.5384 |
| 1 | rs1583377 | 0.5399 | 0.4883 | 0.6036 | 0.555 |
| 6 | rs2395033 | 0.5399 | 0.5904 | 0.4973 | 0.5366 |
| 3 | rs16827475 | 0.5398 | 0.5452 | 0.5345 | 0.539 |
| 7 | rs17533461 | 0.5398 | 0.5948 | 0.4942 | 0.5366 |
| 1 | rs1724951 | 0.5398 | 0.5219 | 0.559 | 0.5433 |
| 16 | rs2892489 | 0.5397 | 0.5627 | 0.5186 | 0.5372 |
| 6 | rs7761214 | 0.5397 | 0.5364 | 0.543 | 0.5403 |
| 6 | rs816374 | 0.5397 | 0.5364 | 0.543 | 0.5403 |
| 6 | rs2856717 | 0.5397 | 0.6341 | 0.4697 | 0.539 |
| 18 | rs2290871 | 0.5396 | 0.5714 | 0.5112 | 0.5366 |
| 6 | rs7192 | 0.5395 | 0.6356 | 0.4687 | 0.539 |
| 18 | rs10468923 | 0.5395 | 0.5685 | 0.5133 | 0.5366 |
| 10 | rs10509011 | 0.5395 | 0.5685 | 0.5133 | 0.5366 |
| 1 | rs11806028 | 0.5394 | 0.5131 | 0.5685 | 0.5452 |
| 15 | rs11634555 | 0.5393 | 0.586 | 0.4995 | 0.536 |
| 6 | rs6935488 | 0.5392 | 0.5102 | 0.5717 | 0.5458 |
| 1 | rs4650353 | 0.5392 | 0.5102 | 0.5717 | 0.5458 |
| 2 | rs1190116 | 0.5392 | 0.5496 | 0.5292 | 0.5378 |
| 6 | rs4945768 | 0.5391 | 0.5292 | 0.5494 | 0.5409 |
| 6 | rs2844713 | 0.5391 | 0.5977 | 0.491 | 0.536 |
| 1 | rs11209032 | 0.5391 | 0.4942 | 0.593 | 0.5513 |
| 14 | rs9888615 | 0.5391 | 0.5729 | 0.509 | 0.536 |
| 1 | rs10128020 | 0.5391 | 0.5233 | 0.5558 | 0.5421 |
| 1 | rs1482160 | 0.5391 | 0.5233 | 0.5558 | 0.5421 |
| 6 | rs708038 | 0.539 | 0.5991 | 0.4899 | 0.536 |
| 6 | rs7772549 | 0.539 | 0.4708 | 0.6302 | 0.563 |
| 7 | rs7806442 | 0.5389 | 0.5131 | 0.5675 | 0.5446 |
| 2 | rs7572996 | 0.5389 | 0.5685 | 0.5122 | 0.536 |
| 12 | rs1798616 | 0.5389 | 0.484 | 0.6079 | 0.5556 |
| 2 | rs9989794 | 0.5388 | 0.5671 | 0.5133 | 0.536 |
| 13 | rs7325396 | 0.5388 | 0.5306 | 0.5473 | 0.5403 |
| 11 | rs3740861 | 0.5388 | 0.5656 | 0.5143 | 0.536 |
| 13 | rs530240 | 0.5388 | 0.5656 | 0.5143 | 0.536 |
| 7 | rs2240851 | 0.5387 | 0.5408 | 0.5367 | 0.5384 |
| 3 | rs17678033 | 0.5387 | 0.5044 | 0.5781 | 0.547 |
| 17 | rs4791927 | 0.5387 | 0.519 | 0.56 | 0.5427 |
| 20 | rs6048350 | 0.5387 | 0.5641 | 0.5154 | 0.536 |
| 4 | rs284793 | 0.5387 | 0.5845 | 0.4995 | 0.5353 |
| 19 | rs12459008 | 0.5387 | 0.6166 | 0.4782 | 0.5366 |
| 6 | rs6912925 | 0.5387 | 0.6166 | 0.4782 | 0.5366 |
| 6 | rs9468926 | 0.5387 | 0.5831 | 0.5005 | 0.5353 |
| 7 | rs2714872 | 0.5387 | 0.5831 | 0.5005 | 0.5353 |
| 2 | rs3109320 | 0.5387 | 0.5831 | 0.5005 | 0.5353 |
| 12 | rs7484827 | 0.5386 | 0.5554 | 0.5228 | 0.5366 |
| 14 | rs6574463 | 0.5386 | 0.4985 | 0.5855 | 0.5489 |
| 11 | rs4537730 | 0.5385 | 0.5204 | 0.5579 | 0.5421 |
| 9 | rs870652 | 0.5385 | 0.5481 | 0.5292 | 0.5372 |
| 8 | rs6987005 | 0.5384 | 0.5087 | 0.5717 | 0.5452 |
| 14 | rs4982026 | 0.5384 | 0.5423 | 0.5345 | 0.5378 |
| 15 | rs17714837 | 0.5384 | 0.5525 | 0.525 | 0.5366 |
| 15 | rs4966035 | 0.5384 | 0.57 | 0.5101 | 0.5353 |
| 8 | rs2645415 | 0.5383 | 0.5685 | 0.5112 | 0.5353 |
| 7 | rs12718939 | 0.5383 | 0.5219 | 0.5558 | 0.5415 |
| 12 | rs11045338 | 0.5383 | 0.5583 | 0.5197 | 0.536 |
| 16 | rs4782578 | 0.5383 | 0.5044 | 0.577 | 0.5464 |
| 5 | rs9687411 | 0.5382 | 0.5335 | 0.543 | 0.539 |
| 11 | rs10836708 | 0.5382 | 0.5335 | 0.543 | 0.539 |
| 16 | rs2178724 | 0.5382 | 0.551 | 0.526 | 0.5366 |
| 5 | rs973149 | 0.5382 | 0.519 | 0.559 | 0.5421 |
| 6 | rs2428501 | 0.5382 | 0.519 | 0.559 | 0.5421 |
| 1 | rs4078266 | 0.5382 | 0.4796 | 0.6132 | 0.5569 |
| 11 | rs16929946 | 0.5382 | 0.4927 | 0.593 | 0.5507 |
| 7 | rs13231224 | 0.5382 | 0.5569 | 0.5207 | 0.536 |
| 17 | rs1420803 | 0.5381 | 0.4971 | 0.5866 | 0.5489 |
| 17 | rs2463496 | 0.5381 | 0.5641 | 0.5143 | 0.5353 |
| 3 | rs1656387 | 0.5381 | 0.5292 | 0.5473 | 0.5396 |
| 1 | rs12043515 | 0.5381 | 0.5233 | 0.5537 | 0.5409 |
| 1 | rs1573738 | 0.5381 | 0.5073 | 0.5728 | 0.5452 |
| 1 | rs1573738 | 0.5381 | 0.5073 | 0.5728 | 0.5452 |
| 10 | rs650058 | 0.538 | 0.5816 | 0.5005 | 0.5347 |
| 11 | rs10836729 | 0.538 | 0.5802 | 0.5016 | 0.5347 |
| 6 | rs3132468 | 0.538 | 0.5889 | 0.4952 | 0.5347 |
| 5 | rs2299009 | 0.538 | 0.5204 | 0.5569 | 0.5415 |
| 1 | rs1339729 | 0.538 | 0.5204 | 0.5569 | 0.5415 |
| 13 | rs9561466 | 0.538 | 0.5394 | 0.5367 | 0.5378 |
| 8 | rs6577853 | 0.538 | 0.5394 | 0.5367 | 0.5378 |
| 4 | rs11944159 | 0.538 | 0.5758 | 0.5048 | 0.5347 |
| 4 | rs16870839 | 0.5379 | 0.5481 | 0.5282 | 0.5366 |
| 5 | rs11743694 | 0.5379 | 0.5175 | 0.56 | 0.5421 |
| 3 | rs6444848 | 0.5378 | 0.5598 | 0.5175 | 0.5353 |
| 11 | rs7116176 | 0.5378 | 0.5525 | 0.5239 | 0.536 |
| 5 | rs11959818 | 0.5378 | 0.4796 | 0.6121 | 0.5562 |
| 4 | rs17474369 | 0.5378 | 0.5102 | 0.5685 | 0.5439 |
| 12 | rs12229242 | 0.5378 | 0.57 | 0.509 | 0.5347 |
| 10 | rs1756740 | 0.5378 | 0.57 | 0.509 | 0.5347 |
| 16 | rs17648946 | 0.5378 | 0.4927 | 0.5919 | 0.5501 |
| 4 | rs1500463 | 0.5378 | 0.4898 | 0.5962 | 0.5513 |
| 17 | rs9674546 | 0.5378 | 0.4942 | 0.5898 | 0.5495 |
| 17 | rs11079590 | 0.5377 | 0.4956 | 0.5877 | 0.5489 |
| 7 | rs6959690 | 0.5377 | 0.5335 | 0.542 | 0.5384 |
| 11 | rs4910003 | 0.5377 | 0.5058 | 0.5739 | 0.5452 |
| 13 | rs4338681 | 0.5377 | 0.551 | 0.525 | 0.536 |
| 10 | rs10764806 | 0.5377 | 0.5117 | 0.5664 | 0.5433 |
| 6 | rs6933779 | 0.5376 | 0.6735 | 0.4474 | 0.5427 |
| 9 | rs944582 | 0.5376 | 0.5364 | 0.5388 | 0.5378 |
| 4 | rs17534458 | 0.5376 | 0.5364 | 0.5388 | 0.5378 |
| 1 | rs504242 | 0.5376 | 0.5262 | 0.5494 | 0.5396 |
| 10 | rs2394527 | 0.5376 | 0.4825 | 0.6068 | 0.5544 |
| 19 | rs8110833 | 0.5376 | 0.5292 | 0.5462 | 0.539 |
| 19 | rs11084211 | 0.5375 | 0.6283 | 0.4697 | 0.5366 |
| 13 | rs2324341 | 0.5375 | 0.5554 | 0.5207 | 0.5353 |
| 16 | rs1563655 | 0.5375 | 0.5321 | 0.543 | 0.5384 |
| 1 | rs902438 | 0.5375 | 0.5087 | 0.5696 | 0.5439 |
| 6 | rs17086323 | 0.5374 | 0.5831 | 0.4984 | 0.5341 |
| 16 | rs2927323 | 0.5374 | 0.5627 | 0.5143 | 0.5347 |
| 11 | rs10830963 | 0.5374 | 0.535 | 0.5399 | 0.5378 |
| 20 | rs6070846 | 0.5374 | 0.5481 | 0.5271 | 0.536 |
| 3 | rs12636098 | 0.5374 | 0.5773 | 0.5027 | 0.5341 |
| 5 | rs11750933 | 0.5374 | 0.5758 | 0.5037 | 0.5341 |
| 18 | rs7505196 | 0.5373 | 0.5146 | 0.5622 | 0.5421 |
| 6 | rs9263871 | 0.5373 | 0.6166 | 0.4761 | 0.5353 |
| 10 | rs11101565 | 0.5373 | 0.5423 | 0.5324 | 0.5366 |
| 15 | rs12899715 | 0.5372 | 0.5598 | 0.5165 | 0.5347 |
| 17 | rs12451047 | 0.5372 | 0.5714 | 0.5069 | 0.5341 |
| 10 | rs4881260 | 0.5372 | 0.5058 | 0.5728 | 0.5446 |
| 1 | rs11581667 | 0.5372 | 0.5058 | 0.5728 | 0.5446 |
| 5 | rs1563510 | 0.5372 | 0.5058 | 0.5728 | 0.5446 |
| 1 | rs7553229 | 0.5372 | 0.5335 | 0.5409 | 0.5378 |
| 2 | rs10432500 | 0.5372 | 0.5117 | 0.5654 | 0.5427 |
| 6 | rs2844580 | 0.5372 | 0.6254 | 0.4708 | 0.536 |
| X | rs6610933 | 0.5371 | 0.5583 | 0.5175 | 0.5347 |
| 10 | rs7897562 | 0.5371 | 0.4825 | 0.6057 | 0.5538 |
| 1 | rs4648482 | 0.5371 | 0.5671 | 0.5101 | 0.5341 |
| 1 | rs4648482 | 0.5371 | 0.5671 | 0.5101 | 0.5341 |
| 7 | rs7777727 | 0.5371 | 0.5233 | 0.5515 | 0.5396 |
| 4 | rs4699559 | 0.537 | 0.5204 | 0.5547 | 0.5403 |
| 8 | rs12676916 | 0.537 | 0.5204 | 0.5547 | 0.5403 |
| 6 | rs2856993 | 0.537 | 0.6691 | 0.4485 | 0.5415 |
| 7 | rs6968514 | 0.537 | 0.5087 | 0.5685 | 0.5433 |
| 1 | rs829417 | 0.537 | 0.5321 | 0.542 | 0.5378 |
| 1 | rs829417 | 0.537 | 0.5321 | 0.542 | 0.5378 |
| 19 | rs330877 | 0.5369 | 0.5554 | 0.5197 | 0.5347 |
| 1 | rs714576 | 0.5369 | 0.5641 | 0.5122 | 0.5341 |
| 8 | rs831725 | 0.5369 | 0.5437 | 0.5303 | 0.536 |
| 6 | rs2074504 | 0.5369 | 0.5437 | 0.5303 | 0.536 |
| 13 | rs17060285 | 0.5369 | 0.5437 | 0.5303 | 0.536 |
| 9 | rs13287131 | 0.5369 | 0.4883 | 0.5962 | 0.5507 |
| 9 | rs1780464 | 0.5369 | 0.4781 | 0.6121 | 0.5556 |
| 6 | rs2857201 | 0.5369 | 0.535 | 0.5388 | 0.5372 |
| 17 | rs16957702 | 0.5369 | 0.535 | 0.5388 | 0.5372 |
| 6 | rs947340 | 0.5368 | 0.5481 | 0.526 | 0.5353 |
| 22 | rs4823168 | 0.5368 | 0.5481 | 0.526 | 0.5353 |
| 11 | rs3132810 | 0.5368 | 0.5146 | 0.5611 | 0.5415 |
| 5 | rs17581793 | 0.5368 | 0.5146 | 0.5611 | 0.5415 |
| 3 | rs4685750 | 0.5368 | 0.5146 | 0.5611 | 0.5415 |
| 8 | rs7006495 | 0.5368 | 0.5845 | 0.4963 | 0.5335 |
| 2 | rs13021276 | 0.5368 | 0.5802 | 0.4995 | 0.5335 |
| 14 | rs2626410 | 0.5368 | 0.586 | 0.4952 | 0.5335 |
| 6 | rs9368002 | 0.5368 | 0.5219 | 0.5526 | 0.5396 |
| 4 | rs1466215 | 0.5368 | 0.5219 | 0.5526 | 0.5396 |
| 4 | rs11726181 | 0.5368 | 0.5058 | 0.5717 | 0.5439 |
| 10 | rs10823282 | 0.5368 | 0.5612 | 0.5143 | 0.5341 |
| 6 | rs683456 | 0.5368 | 0.5423 | 0.5314 | 0.536 |
| 6 | rs6457736 | 0.5367 | 0.5758 | 0.5027 | 0.5335 |
| 6 | rs1265109 | 0.5367 | 0.4985 | 0.5813 | 0.5464 |
| 15 | rs12916262 | 0.5367 | 0.4985 | 0.5813 | 0.5464 |
| 11 | rs12788404 | 0.5367 | 0.5379 | 0.5356 | 0.5366 |
| 8 | rs6983640 | 0.5367 | 0.519 | 0.5558 | 0.5403 |
| 6 | rs6458155 | 0.5367 | 0.5117 | 0.5643 | 0.5421 |
| 14 | rs28607202 | 0.5367 | 0.5729 | 0.5048 | 0.5335 |
| 15 | rs2303310 | 0.5367 | 0.5 | 0.5792 | 0.5458 |
| 6 | rs3132613 | 0.5367 | 0.5598 | 0.5154 | 0.5341 |
| 16 | rs11149623 | 0.5367 | 0.5335 | 0.5399 | 0.5372 |
| 5 | rs10462596 | 0.5367 | 0.5933 | 0.4899 | 0.5335 |
| 18 | rs482570 | 0.5366 | 0.4796 | 0.6089 | 0.5544 |
| 11 | rs10838207 | 0.5366 | 0.551 | 0.5228 | 0.5347 |
| 15 | rs597414 | 0.5366 | 0.551 | 0.5228 | 0.5347 |
| 11 | rs1214810 | 0.5366 | 0.5233 | 0.5505 | 0.539 |
| 7 | rs1525830 | 0.5365 | 0.5685 | 0.508 | 0.5335 |
| 10 | rs5030949 | 0.5365 | 0.5292 | 0.5441 | 0.5378 |
| 15 | rs1875417 | 0.5365 | 0.5204 | 0.5537 | 0.5396 |
| 5 | rs7709606 | 0.5365 | 0.5204 | 0.5537 | 0.5396 |
| 9 | rs10114615 | 0.5365 | 0.5087 | 0.5675 | 0.5427 |
| 11 | rs17651598 | 0.5365 | 0.5321 | 0.5409 | 0.5372 |
| 2 | rs2679853 | 0.5365 | 0.5175 | 0.5569 | 0.5403 |
| 7 | rs10085897 | 0.5365 | 0.5175 | 0.5569 | 0.5403 |
| 6 | rs2621373 | 0.5364 | 0.4767 | 0.6132 | 0.5556 |
| 4 | rs4692477 | 0.5364 | 0.5554 | 0.5186 | 0.5341 |
| 10 | rs857373 | 0.5363 | 0.5641 | 0.5112 | 0.5335 |
| 5 | rs159349 | 0.5363 | 0.5481 | 0.525 | 0.5347 |
| 1 | rs2071956 | 0.5363 | 0.5481 | 0.525 | 0.5347 |
| 1 | rs2071956 | 0.5363 | 0.5481 | 0.525 | 0.5347 |
| 2 | rs12475640 | 0.5363 | 0.5277 | 0.5452 | 0.5378 |
| 21 | rs2838358 | 0.5363 | 0.5277 | 0.5452 | 0.5378 |
| 6 | rs2073010 | 0.5363 | 0.5627 | 0.5122 | 0.5335 |
| 11 | rs863731 | 0.5363 | 0.5539 | 0.5197 | 0.5341 |
| 4 | rs1378945 | 0.5363 | 0.5539 | 0.5197 | 0.5341 |
| 6 | rs2797429 | 0.5362 | 0.5306 | 0.542 | 0.5372 |
| 9 | rs7852684 | 0.5362 | 0.5423 | 0.5303 | 0.5353 |
| 16 | rs1563654 | 0.5362 | 0.5423 | 0.5303 | 0.5353 |
| 21 | rs8132194 | 0.5362 | 0.5423 | 0.5303 | 0.5353 |
| 8 | rs2196904 | 0.5362 | 0.5816 | 0.4973 | 0.5329 |
| 12 | rs4761594 | 0.5362 | 0.5845 | 0.4952 | 0.5329 |
| 6 | rs929160 | 0.5362 | 0.5875 | 0.4931 | 0.5329 |
| 10 | rs10887536 | 0.5361 | 0.5466 | 0.526 | 0.5347 |
| 6 | rs3130041 | 0.5361 | 0.5758 | 0.5016 | 0.5329 |
| 4 | rs7442176 | 0.5361 | 0.5889 | 0.492 | 0.5329 |
| 3 | rs1286654 | 0.5361 | 0.5743 | 0.5027 | 0.5329 |
| 9 | rs10114665 | 0.5361 | 0.5743 | 0.5027 | 0.5329 |
| 13 | rs7983595 | 0.5361 | 0.5743 | 0.5027 | 0.5329 |
| 1 | rs4494160 | 0.5361 | 0.5598 | 0.5143 | 0.5335 |
| 2 | rs10490656 | 0.5361 | 0.5918 | 0.4899 | 0.5329 |
| 1 | rs2088828 | 0.5361 | 0.5233 | 0.5494 | 0.5384 |
| 1 | rs2088828 | 0.5361 | 0.5233 | 0.5494 | 0.5384 |
| 5 | rs7719651 | 0.536 | 0.5292 | 0.543 | 0.5372 |
| 11 | rs2845624 | 0.536 | 0.57 | 0.5058 | 0.5329 |
| 10 | rs7078809 | 0.536 | 0.57 | 0.5058 | 0.5329 |
| 15 | rs4965602 | 0.536 | 0.5452 | 0.5271 | 0.5347 |
| 9 | rs17176112 | 0.536 | 0.5175 | 0.5558 | 0.5396 |
| 15 | rs893816 | 0.5359 | 0.5044 | 0.5717 | 0.5433 |
| 7 | rs7799661 | 0.5359 | 0.6239 | 0.4697 | 0.5347 |
| 21 | rs2249057 | 0.5359 | 0.5321 | 0.5399 | 0.5366 |
| 5 | rs7717058 | 0.5359 | 0.5569 | 0.5165 | 0.5335 |
| 18 | rs12456790 | 0.5359 | 0.5496 | 0.5228 | 0.5341 |
| 9 | rs633903 | 0.5359 | 0.5977 | 0.4857 | 0.5329 |
| 5 | rs903380 | 0.5359 | 0.5977 | 0.4857 | 0.5329 |
| 11 | rs4755797 | 0.5358 | 0.5219 | 0.5505 | 0.5384 |
| 6 | rs2857106 | 0.5358 | 0.6676 | 0.4474 | 0.5403 |
| 6 | rs1736919 | 0.5357 | 0.519 | 0.5537 | 0.539 |
| 10 | rs7092091 | 0.5357 | 0.519 | 0.5537 | 0.539 |
| 6 | rs4441945 | 0.5357 | 0.5481 | 0.5239 | 0.5341 |
| 2 | rs10195293 | 0.5357 | 0.5306 | 0.5409 | 0.5366 |
| 7 | rs2237435 | 0.5357 | 0.5539 | 0.5186 | 0.5335 |
| 5 | rs9312746 | 0.5357 | 0.5015 | 0.5749 | 0.5439 |
| 7 | rs45 | 0.5357 | 0.5379 | 0.5335 | 0.5353 |
| 10 | rs1335701 | 0.5356 | 0.4898 | 0.5909 | 0.5482 |
| 3 | rs950753 | 0.5356 | 0.4869 | 0.5951 | 0.5495 |
| 15 | rs2584180 | 0.5356 | 0.5802 | 0.4973 | 0.5323 |
| 15 | rs4887017 | 0.5356 | 0.5087 | 0.5654 | 0.5415 |
| 20 | rs928163 | 0.5355 | 0.5233 | 0.5484 | 0.5378 |
| 11 | rs514921 | 0.5355 | 0.5758 | 0.5005 | 0.5323 |
| 17 | rs9894659 | 0.5355 | 0.5875 | 0.492 | 0.5323 |
| 11 | rs4910059 | 0.5355 | 0.5889 | 0.491 | 0.5323 |
| 7 | rs13243235 | 0.5355 | 0.5408 | 0.5303 | 0.5347 |
| 2 | rs11902680 | 0.5355 | 0.5364 | 0.5345 | 0.5353 |
| 4 | rs17429224 | 0.5355 | 0.5729 | 0.5027 | 0.5323 |
| 18 | rs10514019 | 0.5355 | 0.5729 | 0.5027 | 0.5323 |
| 16 | rs9922943 | 0.5355 | 0.4621 | 0.6366 | 0.563 |
| 13 | rs9524362 | 0.5354 | 0.5452 | 0.526 | 0.5341 |
| 11 | rs11600666 | 0.5354 | 0.5321 | 0.5388 | 0.536 |
| 8 | rs12546089 | 0.5354 | 0.4985 | 0.5781 | 0.5446 |
| X | rs982767 | 0.5354 | 0.4985 | 0.5781 | 0.5446 |
| 9 | rs1986863 | 0.5354 | 0.5146 | 0.5579 | 0.5396 |
| 7 | rs2141780 | 0.5353 | 0.5569 | 0.5154 | 0.5329 |
| 11 | rs1144746 | 0.5353 | 0.5569 | 0.5154 | 0.5329 |
| 10 | rs10881975 | 0.5353 | 0.5569 | 0.5154 | 0.5329 |
| 8 | rs726723 | 0.5353 | 0.5569 | 0.5154 | 0.5329 |
| 5 | rs26291 | 0.5353 | 0.5 | 0.576 | 0.5439 |
| 10 | rs10906416 | 0.5353 | 0.5671 | 0.5069 | 0.5323 |
| 9 | rs7031744 | 0.5353 | 0.5671 | 0.5069 | 0.5323 |
| 2 | rs12613538 | 0.5353 | 0.5248 | 0.5462 | 0.5372 |
| 7 | rs805787 | 0.5353 | 0.5219 | 0.5494 | 0.5378 |
| 1 | rs3131514 | 0.5353 | 0.5277 | 0.543 | 0.5366 |
| 1 | rs3131514 | 0.5353 | 0.5277 | 0.543 | 0.5366 |
| 2 | rs13411813 | 0.5353 | 0.5277 | 0.543 | 0.5366 |
| 6 | rs1233367 | 0.5352 | 0.5117 | 0.5611 | 0.5403 |
| 10 | rs10886463 | 0.5352 | 0.5656 | 0.508 | 0.5323 |
| 6 | rs2495990 | 0.5352 | 0.5977 | 0.4846 | 0.5323 |
| 1 | rs12746015 | 0.5352 | 0.516 | 0.5558 | 0.539 |
| 10 | rs10787924 | 0.5352 | 0.516 | 0.5558 | 0.539 |
| X | rs12216952 | 0.5352 | 0.4767 | 0.61 | 0.5538 |
| 6 | rs2523870 | 0.5351 | 0.6181 | 0.4718 | 0.5335 |
| 2 | rs7580177 | 0.5351 | 0.5379 | 0.5324 | 0.5347 |
| 13 | rs9571745 | 0.5351 | 0.6006 | 0.4825 | 0.5323 |
| 9 | rs10814119 | 0.535 | 0.5233 | 0.5473 | 0.5372 |
| 2 | rs923334 | 0.535 | 0.5466 | 0.5239 | 0.5335 |
| 18 | rs11081604 | 0.535 | 0.5466 | 0.5239 | 0.5335 |
| 4 | rs3733190 | 0.535 | 0.5612 | 0.5112 | 0.5323 |
| 7 | rs10265287 | 0.535 | 0.5612 | 0.5112 | 0.5323 |
| 11 | rs492504 | 0.535 | 0.5262 | 0.5441 | 0.5366 |
| 8 | rs1043720 | 0.535 | 0.5292 | 0.5409 | 0.536 |
| 6 | rs9466456 | 0.535 | 0.5408 | 0.5292 | 0.5341 |
| 6 | rs199059 | 0.535 | 0.5364 | 0.5335 | 0.5347 |
| 18 | rs12605134 | 0.535 | 0.5364 | 0.5335 | 0.5347 |
| 15 | rs11857332 | 0.535 | 0.5364 | 0.5335 | 0.5347 |
| 20 | rs208380 | 0.535 | 0.5787 | 0.4973 | 0.5317 |
| 6 | rs1610602 | 0.5349 | 0.5845 | 0.4931 | 0.5317 |
| 2 | rs13017308 | 0.5349 | 0.5102 | 0.5622 | 0.5403 |
| 5 | rs1809814 | 0.5349 | 0.5102 | 0.5622 | 0.5403 |
| X | rs5924293 | 0.5349 | 0.5758 | 0.4995 | 0.5317 |
| 16 | rs11642469 | 0.5349 | 0.5743 | 0.5005 | 0.5317 |
| 16 | rs7198575 | 0.5349 | 0.5743 | 0.5005 | 0.5317 |
| X | rs6579495 | 0.5349 | 0.5875 | 0.491 | 0.5317 |
| 3 | rs3934936 | 0.5349 | 0.5146 | 0.5569 | 0.539 |
| 4 | rs11096964 | 0.5349 | 0.5729 | 0.5016 | 0.5317 |
| 18 | rs8096793 | 0.5349 | 0.5583 | 0.5133 | 0.5323 |
| 7 | rs11770859 | 0.5348 | 0.57 | 0.5037 | 0.5317 |
| PAR | rs611711 | 0.5348 | 0.5918 | 0.4878 | 0.5317 |
| 6 | rs2507984 | 0.5348 | 0.5394 | 0.5303 | 0.5341 |
| 6 | rs6939576 | 0.5348 | 0.5394 | 0.5303 | 0.5341 |
| 9 | rs6597539 | 0.5348 | 0.5219 | 0.5484 | 0.5372 |
| 8 | rs7814793 | 0.5348 | 0.5117 | 0.56 | 0.5396 |
| 11 | rs1387153 | 0.5348 | 0.5569 | 0.5143 | 0.5323 |
| 7 | rs1031276 | 0.5348 | 0.5685 | 0.5048 | 0.5317 |
| 8 | rs4876542 | 0.5348 | 0.5496 | 0.5207 | 0.5329 |
| 6 | rs6907236 | 0.5348 | 0.6283 | 0.4655 | 0.5341 |
| 11 | rs10750225 | 0.5348 | 0.5073 | 0.5654 | 0.5409 |
| 5 | rs26997 | 0.5348 | 0.5073 | 0.5654 | 0.5409 |
| 10 | rs2960660 | 0.5348 | 0.5073 | 0.5654 | 0.5409 |
| 11 | rs10836061 | 0.5348 | 0.519 | 0.5515 | 0.5378 |
| 16 | rs8053253 | 0.5347 | 0.5933 | 0.4867 | 0.5317 |
| 14 | rs7160581 | 0.5347 | 0.5277 | 0.542 | 0.536 |
| 16 | rs363157 | 0.5347 | 0.4913 | 0.5866 | 0.5464 |
| 6 | rs1116221 | 0.5347 | 0.5671 | 0.5058 | 0.5317 |
| 3 | rs1286750 | 0.5347 | 0.5554 | 0.5154 | 0.5323 |
| 20 | rs3787555 | 0.5346 | 0.5962 | 0.4846 | 0.5317 |
| 9 | rs1576676 | 0.5346 | 0.5481 | 0.5218 | 0.5329 |
| 6 | rs2754809 | 0.5346 | 0.5481 | 0.5218 | 0.5329 |
| 8 | rs7828292 | 0.5346 | 0.5379 | 0.5314 | 0.5341 |
| 4 | rs17710575 | 0.5346 | 0.5379 | 0.5314 | 0.5341 |
| 7 | rs1364733 | 0.5346 | 0.5087 | 0.5632 | 0.5403 |
| 16 | rs11074782 | 0.5346 | 0.5335 | 0.5356 | 0.5347 |
| 1 | rs10799812 | 0.5345 | 0.5204 | 0.5494 | 0.5372 |
| 1 | rs10799812 | 0.5345 | 0.5204 | 0.5494 | 0.5372 |
| 7 | rs7778976 | 0.5345 | 0.5627 | 0.509 | 0.5317 |
| 8 | rs715806 | 0.5345 | 0.5262 | 0.543 | 0.536 |
| 6 | rs6933014 | 0.5344 | 0.5292 | 0.5399 | 0.5353 |
| 3 | rs7649882 | 0.5344 | 0.5292 | 0.5399 | 0.5353 |
| X | rs10482291 | 0.5344 | 0.5612 | 0.5101 | 0.5317 |
| 2 | rs6719068 | 0.5344 | 0.5612 | 0.5101 | 0.5317 |
| 9 | rs1551411 | 0.5344 | 0.5612 | 0.5101 | 0.5317 |
| 18 | rs2850698 | 0.5344 | 0.4781 | 0.6057 | 0.5519 |
| 8 | rs2673570 | 0.5344 | 0.5364 | 0.5324 | 0.5341 |
| 12 | rs2731428 | 0.5344 | 0.5408 | 0.5282 | 0.5335 |
| 15 | rs12915037 | 0.5344 | 0.5408 | 0.5282 | 0.5335 |
| 2 | rs2863301 | 0.5344 | 0.5146 | 0.5558 | 0.5384 |
| 3 | rs9865620 | 0.5344 | 0.5146 | 0.5558 | 0.5384 |
| 10 | rs2960680 | 0.5344 | 0.5 | 0.5739 | 0.5427 |
| 15 | rs1345919 | 0.5344 | 0.465 | 0.6281 | 0.5593 |
| 4 | rs1458893 | 0.5344 | 0.5598 | 0.5112 | 0.5317 |
| 5 | rs270192 | 0.5343 | 0.5802 | 0.4952 | 0.531 |
| 15 | rs7178674 | 0.5343 | 0.5802 | 0.4952 | 0.531 |
| 3 | rs4591526 | 0.5343 | 0.5452 | 0.5239 | 0.5329 |
| 6 | rs28730974 | 0.5343 | 0.5816 | 0.4942 | 0.531 |
| 12 | rs12312778 | 0.5343 | 0.4767 | 0.6079 | 0.5526 |
| 6 | rs3094188 | 0.5343 | 0.5831 | 0.4931 | 0.531 |
| 5 | rs2891894 | 0.5343 | 0.5831 | 0.4931 | 0.531 |
| 6 | rs4145451 | 0.5343 | 0.6195 | 0.4697 | 0.5329 |
| 2 | rs13420053 | 0.5343 | 0.551 | 0.5186 | 0.5323 |
| 1 | rs947647 | 0.5343 | 0.551 | 0.5186 | 0.5323 |
| 3 | rs17043256 | 0.5343 | 0.5758 | 0.4984 | 0.531 |
| 5 | rs17802003 | 0.5343 | 0.5758 | 0.4984 | 0.531 |
| 4 | rs1318822 | 0.5343 | 0.5845 | 0.492 | 0.531 |
| 1 | rs491513 | 0.5343 | 0.5219 | 0.5473 | 0.5366 |
| 15 | rs12900283 | 0.5343 | 0.5117 | 0.559 | 0.539 |
| 10 | rs10509636 | 0.5343 | 0.5583 | 0.5122 | 0.5317 |
| 9 | rs17802846 | 0.5343 | 0.5729 | 0.5005 | 0.531 |
| 9 | rs5018269 | 0.5343 | 0.5729 | 0.5005 | 0.531 |
| 8 | rs7013253 | 0.5343 | 0.4854 | 0.594 | 0.5482 |
| 16 | rs1034392 | 0.5343 | 0.5248 | 0.5441 | 0.536 |
| 9 | rs7869768 | 0.5343 | 0.519 | 0.5505 | 0.5372 |
| 1 | rs7556364 | 0.5342 | 0.4927 | 0.5834 | 0.5452 |
| 7 | rs13235075 | 0.5342 | 0.5394 | 0.5292 | 0.5335 |
| 2 | rs1349520 | 0.5342 | 0.4942 | 0.5813 | 0.5446 |
| 1 | rs6703022 | 0.5342 | 0.57 | 0.5027 | 0.531 |
| 18 | rs10871746 | 0.5342 | 0.5437 | 0.525 | 0.5329 |
| 8 | rs2553651 | 0.5342 | 0.5029 | 0.5696 | 0.5415 |
| 5 | rs16899370 | 0.5342 | 0.5437 | 0.525 | 0.5329 |
| 6 | rs2517552 | 0.5342 | 0.621 | 0.4687 | 0.5329 |
| 1 | rs3767141 | 0.5341 | 0.5306 | 0.5377 | 0.5347 |
| 1 | rs3767141 | 0.5341 | 0.5306 | 0.5377 | 0.5347 |
| 21 | rs2830500 | 0.5341 | 0.5554 | 0.5143 | 0.5317 |
| 5 | rs10071234 | 0.5341 | 0.5044 | 0.5675 | 0.5409 |
| 16 | rs11861743 | 0.5341 | 0.5481 | 0.5207 | 0.5323 |
| 9 | rs10114292 | 0.5341 | 0.5656 | 0.5058 | 0.531 |
| 6 | rs3130636 | 0.5341 | 0.5948 | 0.4846 | 0.531 |
| 9 | rs12004667 | 0.534 | 0.5423 | 0.526 | 0.5329 |
| 9 | rs10821326 | 0.534 | 0.5335 | 0.5345 | 0.5341 |
| 9 | rs10122524 | 0.534 | 0.5335 | 0.5345 | 0.5341 |
| 17 | rs10521218 | 0.534 | 0.5335 | 0.5345 | 0.5341 |
| 7 | rs1568658 | 0.534 | 0.5233 | 0.5452 | 0.536 |
| 4 | rs12502935 | 0.534 | 0.5102 | 0.56 | 0.539 |
| 3 | rs7626381 | 0.534 | 0.5102 | 0.56 | 0.539 |
| 8 | rs10956429 | 0.5339 | 0.5 | 0.5728 | 0.5421 |
| 14 | rs11625323 | 0.5339 | 0.5 | 0.5728 | 0.5421 |
| 6 | rs3094562 | 0.5339 | 0.5 | 0.5728 | 0.5421 |
| 6 | rs2517646 | 0.5339 | 0.4592 | 0.6376 | 0.5624 |
| 4 | rs7665110 | 0.5339 | 0.5525 | 0.5165 | 0.5317 |
| 6 | rs13197045 | 0.5339 | 0.5408 | 0.5271 | 0.5329 |
| 19 | rs7507133 | 0.5339 | 0.6239 | 0.4665 | 0.5329 |
| 6 | rs2394660 | 0.5338 | 0.5015 | 0.5707 | 0.5415 |
| 22 | rs9306510 | 0.5338 | 0.5015 | 0.5707 | 0.5415 |
| 6 | rs3130922 | 0.5338 | 0.5321 | 0.5356 | 0.5341 |
| 9 | rs10816257 | 0.5338 | 0.5117 | 0.5579 | 0.5384 |
| 11 | rs4998870 | 0.5338 | 0.5452 | 0.5228 | 0.5323 |
| 11 | rs530771 | 0.5338 | 0.5598 | 0.5101 | 0.531 |
| 4 | rs10938787 | 0.5338 | 0.5248 | 0.543 | 0.5353 |
| 9 | rs10977337 | 0.5338 | 0.5248 | 0.543 | 0.5353 |
| 16 | rs4781071 | 0.5338 | 0.5248 | 0.543 | 0.5353 |
| 7 | rs3988090 | 0.5337 | 0.4606 | 0.6344 | 0.5612 |
| 3 | rs192770 | 0.5337 | 0.4811 | 0.5994 | 0.5495 |
| 6 | rs9468922 | 0.5337 | 0.5802 | 0.4942 | 0.5304 |
| 5 | rs6871626 | 0.5337 | 0.5802 | 0.4942 | 0.5304 |
| 4 | rs17601458 | 0.5337 | 0.5773 | 0.4963 | 0.5304 |
| 8 | rs6578027 | 0.5337 | 0.5773 | 0.4963 | 0.5304 |
| 11 | rs7114658 | 0.5337 | 0.5816 | 0.4931 | 0.5304 |
| 12 | rs4499055 | 0.5337 | 0.5831 | 0.492 | 0.5304 |
| 22 | rs6006521 | 0.5337 | 0.5394 | 0.5282 | 0.5329 |
| 13 | rs7323986 | 0.5337 | 0.535 | 0.5324 | 0.5335 |
| 5 | rs162904 | 0.5337 | 0.6414 | 0.457 | 0.5347 |
| 9 | rs17629093 | 0.5337 | 0.516 | 0.5526 | 0.5372 |
| 10 | rs7079639 | 0.5337 | 0.5583 | 0.5112 | 0.531 |
| 8 | rs17358302 | 0.5337 | 0.516 | 0.5526 | 0.5372 |
| 6 | rs1419642 | 0.5337 | 0.586 | 0.4899 | 0.5304 |
| 10 | rs7905568 | 0.5336 | 0.5714 | 0.5005 | 0.5304 |
| 8 | rs4545135 | 0.5336 | 0.4971 | 0.576 | 0.5427 |
| 14 | rs7141959 | 0.5336 | 0.6195 | 0.4687 | 0.5323 |
| 14 | rs8008967 | 0.5336 | 0.5496 | 0.5186 | 0.5317 |
| 8 | rs2467766 | 0.5336 | 0.6122 | 0.4729 | 0.5317 |
| 13 | rs7987131 | 0.5336 | 0.5569 | 0.5122 | 0.531 |
| 17 | rs1585804 | 0.5336 | 0.57 | 0.5016 | 0.5304 |
| 6 | rs2523651 | 0.5336 | 0.5889 | 0.4878 | 0.5304 |
| 2 | rs10193886 | 0.5336 | 0.5044 | 0.5664 | 0.5403 |
| 10 | rs17445328 | 0.5336 | 0.4781 | 0.6036 | 0.5507 |
| 7 | rs7806458 | 0.5336 | 0.4679 | 0.6206 | 0.5562 |
| 6 | rs2844697 | 0.5336 | 0.5685 | 0.5027 | 0.5304 |
| X | rs4825699 | 0.5336 | 0.4985 | 0.5739 | 0.5421 |
| 17 | rs12602618 | 0.5335 | 0.5379 | 0.5292 | 0.5329 |
| 1 | rs11166101 | 0.5335 | 0.4767 | 0.6057 | 0.5513 |
| 5 | rs2431268 | 0.5335 | 0.5233 | 0.5441 | 0.5353 |
| 14 | rs1767426 | 0.5335 | 0.5233 | 0.5441 | 0.5353 |
| 1 | rs1877724 | 0.5335 | 0.5204 | 0.5473 | 0.536 |
| 2 | rs11676303 | 0.5335 | 0.5481 | 0.5197 | 0.5317 |
| 21 | rs9982895 | 0.5335 | 0.5335 | 0.5335 | 0.5335 |
| 1 | rs589962 | 0.5335 | 0.5423 | 0.525 | 0.5323 |
| 4 | rs3796862 | 0.5335 | 0.5423 | 0.525 | 0.5323 |
| 6 | rs6909636 | 0.5335 | 0.621 | 0.4676 | 0.5323 |
| 5 | rs10063408 | 0.5335 | 0.5102 | 0.559 | 0.5384 |
| 5 | rs12651722 | 0.5335 | 0.5102 | 0.559 | 0.5384 |
| X | rs5926516 | 0.5335 | 0.5102 | 0.559 | 0.5384 |
| 12 | rs11110918 | 0.5335 | 0.5262 | 0.5409 | 0.5347 |
| 5 | rs11954063 | 0.5335 | 0.5262 | 0.5409 | 0.5347 |
| 3 | rs9863850 | 0.5335 | 0.4708 | 0.6153 | 0.5544 |
| 2 | rs10210517 | 0.5335 | 0.5058 | 0.5643 | 0.5396 |
| X | rs3126289 | 0.5335 | 0.5933 | 0.4846 | 0.5304 |
| 10 | rs4934089 | 0.5334 | 0.4883 | 0.5877 | 0.5458 |
| 7 | rs10239822 | 0.5334 | 0.4752 | 0.6079 | 0.5519 |
| 14 | rs17783336 | 0.5334 | 0.4898 | 0.5855 | 0.5452 |
| X | rs984970 | 0.5334 | 0.4854 | 0.5919 | 0.547 |
| 4 | rs1265922 | 0.5334 | 0.4898 | 0.5855 | 0.5452 |
| 14 | rs7161281 | 0.5334 | 0.5539 | 0.5143 | 0.531 |
| 12 | rs10848088 | 0.5334 | 0.5292 | 0.5377 | 0.5341 |
| 16 | rs869426 | 0.5334 | 0.5466 | 0.5207 | 0.5317 |
| 6 | rs1610586 | 0.5334 | 0.5015 | 0.5696 | 0.5409 |
| 9 | rs1107117 | 0.5334 | 0.5015 | 0.5696 | 0.5409 |
| 2 | rs6729040 | 0.5333 | 0.5408 | 0.526 | 0.5323 |
| 1 | rs4131408 | 0.5333 | 0.5408 | 0.526 | 0.5323 |
| 8 | rs13255063 | 0.5333 | 0.5117 | 0.5569 | 0.5378 |
| 5 | rs10472329 | 0.5333 | 0.5321 | 0.5345 | 0.5335 |
| 15 | rs11638527 | 0.5333 | 0.5525 | 0.5154 | 0.531 |
| 13 | rs8001644 | 0.5333 | 0.5612 | 0.508 | 0.5304 |
| 13 | rs405677 | 0.5332 | 0.6443 | 0.4548 | 0.5347 |
| 1 | rs2501257 | 0.5332 | 0.5248 | 0.542 | 0.5347 |
| 1 | rs2501257 | 0.5332 | 0.5248 | 0.542 | 0.5347 |
| 20 | rs2024683 | 0.5332 | 0.5598 | 0.509 | 0.5304 |
| 7 | rs1464804 | 0.5332 | 0.5598 | 0.509 | 0.5304 |
| 1 | rs10927459 | 0.5332 | 0.4723 | 0.6121 | 0.5532 |
| 1 | rs10927459 | 0.5332 | 0.4723 | 0.6121 | 0.5532 |
| 4 | rs13108783 | 0.5332 | 0.551 | 0.5165 | 0.531 |
| 15 | rs658141 | 0.5332 | 0.5277 | 0.5388 | 0.5341 |
| 12 | rs4417320 | 0.5332 | 0.5277 | 0.5388 | 0.5341 |
| 22 | rs10439907 | 0.5332 | 0.4971 | 0.5749 | 0.5421 |
| 6 | rs2517448 | 0.5332 | 0.6297 | 0.4623 | 0.5329 |
| 8 | rs240950 | 0.5332 | 0.5087 | 0.56 | 0.5384 |
| 20 | rs4813164 | 0.5332 | 0.5087 | 0.56 | 0.5384 |
| 5 | rs12332473 | 0.5332 | 0.535 | 0.5314 | 0.5329 |
| 2 | rs4670575 | 0.5332 | 0.535 | 0.5314 | 0.5329 |
| 2 | rs11889710 | 0.5331 | 0.5583 | 0.5101 | 0.5304 |
| 7 | rs1723622 | 0.5331 | 0.5802 | 0.4931 | 0.5298 |
| 15 | rs4775282 | 0.5331 | 0.5773 | 0.4952 | 0.5298 |
| 22 | rs139461 | 0.5331 | 0.4767 | 0.6047 | 0.5507 |
| 20 | rs4811766 | 0.5331 | 0.5306 | 0.5356 | 0.5335 |
| 11 | rs7480118 | 0.5331 | 0.5437 | 0.5228 | 0.5317 |
| X | rs5929504 | 0.5331 | 0.5437 | 0.5228 | 0.5317 |
| 13 | rs9508066 | 0.5331 | 0.5437 | 0.5228 | 0.5317 |
| 2 | rs2581047 | 0.5331 | 0.5743 | 0.4973 | 0.5298 |
| 18 | rs2580145 | 0.5331 | 0.5496 | 0.5175 | 0.531 |
| 4 | rs16852274 | 0.5331 | 0.5496 | 0.5175 | 0.531 |
| 10 | rs2395336 | 0.5331 | 0.5845 | 0.4899 | 0.5298 |
| 11 | rs716146 | 0.533 | 0.5569 | 0.5112 | 0.5304 |
| 18 | rs4624267 | 0.533 | 0.5569 | 0.5112 | 0.5304 |
| 2 | rs13414801 | 0.533 | 0.4665 | 0.6217 | 0.5562 |
| 6 | rs1485784 | 0.533 | 0.5204 | 0.5462 | 0.5353 |
| 22 | rs11556482 | 0.533 | 0.5204 | 0.5462 | 0.5353 |
| 5 | rs33423 | 0.533 | 0.5 | 0.5707 | 0.5409 |
| 12 | rs11106418 | 0.533 | 0.57 | 0.5005 | 0.5298 |
| X | rs7058025 | 0.533 | 0.5102 | 0.5579 | 0.5378 |
| 17 | rs312723 | 0.533 | 0.5058 | 0.5632 | 0.539 |
| 13 | rs1157323 | 0.533 | 0.5058 | 0.5632 | 0.539 |
| 12 | rs11608740 | 0.533 | 0.5058 | 0.5632 | 0.539 |
| 16 | rs8060961 | 0.533 | 0.5379 | 0.5282 | 0.5323 |
| 8 | rs16901797 | 0.533 | 0.4883 | 0.5866 | 0.5452 |
| 7 | rs7800411 | 0.533 | 0.4883 | 0.5866 | 0.5452 |
| 20 | rs4813941 | 0.533 | 0.5335 | 0.5324 | 0.5329 |
| 6 | rs2074470 | 0.533 | 0.4898 | 0.5845 | 0.5446 |
| 4 | rs4691121 | 0.533 | 0.5889 | 0.4867 | 0.5298 |
| 9 | rs10961710 | 0.533 | 0.5262 | 0.5399 | 0.5341 |
| 11 | rs11225886 | 0.533 | 0.5262 | 0.5399 | 0.5341 |
| 15 | rs12442954 | 0.5329 | 0.5481 | 0.5186 | 0.531 |
| 7 | rs3801350 | 0.5329 | 0.5423 | 0.5239 | 0.5317 |
| 3 | rs2870518 | 0.5329 | 0.5554 | 0.5122 | 0.5304 |
| 2 | rs4971807 | 0.5329 | 0.5554 | 0.5122 | 0.5304 |
| 5 | rs6882021 | 0.5329 | 0.5904 | 0.4857 | 0.5298 |
| 15 | rs2956236 | 0.5329 | 0.5904 | 0.4857 | 0.5298 |
| 2 | rs12052449 | 0.5329 | 0.5671 | 0.5027 | 0.5298 |
| 12 | rs1543933 | 0.5329 | 0.5146 | 0.5526 | 0.5366 |
| 20 | rs1041274 | 0.5329 | 0.5146 | 0.5526 | 0.5366 |
| 22 | rs2076578 | 0.5329 | 0.4738 | 0.6089 | 0.5519 |
| 6 | rs10947128 | 0.5329 | 0.637 | 0.458 | 0.5335 |
| 21 | rs11089102 | 0.5329 | 0.5292 | 0.5367 | 0.5335 |
| 1 | rs12733180 | 0.5328 | 0.605 | 0.4761 | 0.5304 |
| X | rs2079861 | 0.5328 | 0.6137 | 0.4708 | 0.531 |
| 6 | rs7764227 | 0.5328 | 0.5117 | 0.5558 | 0.5372 |
| 22 | rs7289450 | 0.5328 | 0.5117 | 0.5558 | 0.5372 |
| 4 | rs11946572 | 0.5328 | 0.5641 | 0.5048 | 0.5298 |
| 3 | rs12492444 | 0.5328 | 0.5641 | 0.5048 | 0.5298 |
| 9 | rs4741434 | 0.5328 | 0.5641 | 0.5048 | 0.5298 |
| 19 | rs1008420 | 0.5328 | 0.5364 | 0.5292 | 0.5323 |
| 5 | rs1445845 | 0.5328 | 0.5364 | 0.5292 | 0.5323 |
| 21 | rs1107121 | 0.5328 | 0.4956 | 0.576 | 0.5421 |
| 1 | rs2703998 | 0.5328 | 0.5408 | 0.525 | 0.5317 |
| 1 | rs16863119 | 0.5328 | 0.5408 | 0.525 | 0.5317 |
| 11 | rs17417943 | 0.5328 | 0.5321 | 0.5335 | 0.5329 |
| 2 | rs4953658 | 0.5328 | 0.5627 | 0.5058 | 0.5298 |
| 2 | rs1076594 | 0.5328 | 0.5627 | 0.5058 | 0.5298 |
| 1 | rs2147477 | 0.5328 | 0.5219 | 0.5441 | 0.5347 |
| 12 | rs10746214 | 0.5328 | 0.5219 | 0.5441 | 0.5347 |
| 14 | rs400314 | 0.5328 | 0.5627 | 0.5058 | 0.5298 |
| 4 | rs11946193 | 0.5328 | 0.5219 | 0.5441 | 0.5347 |
| 13 | rs593390 | 0.5328 | 0.5627 | 0.5058 | 0.5298 |
| 17 | rs12941227 | 0.5328 | 0.5219 | 0.5441 | 0.5347 |
| 1 | rs2485662 | 0.5327 | 0.516 | 0.5505 | 0.536 |
| 6 | rs1736913 | 0.5327 | 0.5612 | 0.5069 | 0.5298 |
| 5 | rs440218 | 0.5327 | 0.5087 | 0.559 | 0.5378 |
| 6 | rs644045 | 0.5327 | 0.5452 | 0.5207 | 0.531 |
| 14 | rs17783366 | 0.5327 | 0.5044 | 0.5643 | 0.539 |
| 11 | rs1146200 | 0.5326 | 0.6283 | 0.4623 | 0.5323 |
| 6 | rs319078 | 0.5326 | 0.535 | 0.5303 | 0.5323 |
| 5 | rs272872 | 0.5326 | 0.551 | 0.5154 | 0.5304 |
| 9 | rs13295646 | 0.5326 | 0.551 | 0.5154 | 0.5304 |
| 5 | rs2337020 | 0.5326 | 0.551 | 0.5154 | 0.5304 |
| 4 | rs13145352 | 0.5326 | 0.5394 | 0.526 | 0.5317 |
| X | rs2158209 | 0.5326 | 0.5598 | 0.508 | 0.5298 |
| 16 | rs2764776 | 0.5326 | 0.5598 | 0.508 | 0.5298 |
| 13 | rs4772944 | 0.5326 | 0.5306 | 0.5345 | 0.5329 |
| 7 | rs2192062 | 0.5326 | 0.5306 | 0.5345 | 0.5329 |
| 8 | rs890032 | 0.5325 | 0.4869 | 0.5877 | 0.5452 |
| 2 | rs17390720 | 0.5325 | 0.5991 | 0.4793 | 0.5298 |
| 12 | rs11048788 | 0.5325 | 0.5 | 0.5696 | 0.5403 |
| 4 | rs1884411 | 0.5325 | 0.5 | 0.5696 | 0.5403 |
| 2 | rs4595925 | 0.5325 | 0.5 | 0.5696 | 0.5403 |
| 6 | rs3131018 | 0.5325 | 0.5583 | 0.509 | 0.5298 |
| 13 | rs563925 | 0.5325 | 0.5437 | 0.5218 | 0.531 |
| 5 | rs10060813 | 0.5325 | 0.4898 | 0.5834 | 0.5439 |
| 2 | rs350786 | 0.5325 | 0.6093 | 0.4729 | 0.5304 |
| 12 | rs1548837 | 0.5325 | 0.6093 | 0.4729 | 0.5304 |
| 9 | rs4878619 | 0.5325 | 0.5058 | 0.5622 | 0.5384 |
| 6 | rs45560332 | 0.5325 | 0.5102 | 0.5569 | 0.5372 |
| 12 | rs11175342 | 0.5325 | 0.5102 | 0.5569 | 0.5372 |
| 1 | rs12060631 | 0.5325 | 0.5496 | 0.5165 | 0.5304 |
| 5 | rs13179925 | 0.5325 | 0.5496 | 0.5165 | 0.5304 |
| 6 | rs2170183 | 0.5325 | 0.5233 | 0.542 | 0.5341 |
| 4 | rs17429245 | 0.5325 | 0.5773 | 0.4942 | 0.5292 |
| 13 | rs1597701 | 0.5325 | 0.5802 | 0.492 | 0.5292 |
| 2 | rs11096556 | 0.5325 | 0.5175 | 0.5484 | 0.5353 |
| 10 | rs2253762 | 0.5325 | 0.5175 | 0.5484 | 0.5353 |
| 10 | rs3127087 | 0.5325 | 0.5175 | 0.5484 | 0.5353 |
| 18 | rs1944002 | 0.5325 | 0.5175 | 0.5484 | 0.5353 |
| 8 | rs1965805 | 0.5325 | 0.5743 | 0.4963 | 0.5292 |
| 14 | rs8011702 | 0.5325 | 0.4811 | 0.5962 | 0.5476 |
| 12 | rs7295457 | 0.5325 | 0.6006 | 0.4782 | 0.5298 |
| 10 | rs7075934 | 0.5325 | 0.6006 | 0.4782 | 0.5298 |
| 3 | rs9876709 | 0.5325 | 0.4927 | 0.5792 | 0.5427 |
| 3 | rs9813870 | 0.5324 | 0.5569 | 0.5101 | 0.5298 |
| 12 | rs11177183 | 0.5324 | 0.5379 | 0.5271 | 0.5317 |
| 17 | rs9906150 | 0.5324 | 0.5379 | 0.5271 | 0.5317 |
| 9 | rs12683380 | 0.5324 | 0.5262 | 0.5388 | 0.5335 |
| 9 | rs11103784 | 0.5324 | 0.5335 | 0.5314 | 0.5323 |
| 9 | rs2506686 | 0.5324 | 0.5335 | 0.5314 | 0.5323 |
| 8 | rs4739996 | 0.5324 | 0.5335 | 0.5314 | 0.5323 |
| 9 | rs4977948 | 0.5324 | 0.5015 | 0.5675 | 0.5396 |
| 7 | rs10234732 | 0.5324 | 0.5015 | 0.5675 | 0.5396 |
| 1 | rs6658454 | 0.5324 | 0.5845 | 0.4888 | 0.5292 |
| 14 | rs1469602 | 0.5324 | 0.5845 | 0.4888 | 0.5292 |
| 1 | rs1938355 | 0.5324 | 0.5714 | 0.4984 | 0.5292 |
| 16 | rs7200174 | 0.5324 | 0.4796 | 0.5983 | 0.5482 |
| 18 | rs2625366 | 0.5324 | 0.4942 | 0.577 | 0.5421 |
| 3 | rs1396032 | 0.5324 | 0.6356 | 0.458 | 0.5329 |
| 6 | rs45552931 | 0.5324 | 0.5073 | 0.56 | 0.5378 |
| 3 | rs9818299 | 0.5324 | 0.5073 | 0.56 | 0.5378 |
| 1 | rs17539583 | 0.5323 | 0.4956 | 0.5749 | 0.5415 |
| 1 | rs6691729 | 0.5323 | 0.5029 | 0.5654 | 0.539 |
| X | rs6617632 | 0.5323 | 0.5364 | 0.5282 | 0.5317 |
| 1 | rs6669656 | 0.5323 | 0.5539 | 0.5122 | 0.5298 |
| 5 | rs1309822 | 0.5323 | 0.5539 | 0.5122 | 0.5298 |
| 4 | rs10007283 | 0.5323 | 0.5539 | 0.5122 | 0.5298 |
| 4 | rs11725452 | 0.5323 | 0.5539 | 0.5122 | 0.5298 |
| 3 | rs6766546 | 0.5323 | 0.4971 | 0.5728 | 0.5409 |
| 1 | rs339565 | 0.5323 | 0.5466 | 0.5186 | 0.5304 |
| 16 | rs1366537 | 0.5323 | 0.5466 | 0.5186 | 0.5304 |
| 6 | rs2504288 | 0.5322 | 0.5219 | 0.543 | 0.5341 |
| 2 | rs9288535 | 0.5322 | 0.5219 | 0.543 | 0.5341 |
| 8 | rs17667710 | 0.5322 | 0.5219 | 0.543 | 0.5341 |
| 2 | rs1403335 | 0.5322 | 0.5321 | 0.5324 | 0.5323 |
| 2 | rs4321409 | 0.5322 | 0.5321 | 0.5324 | 0.5323 |
| 14 | rs12433360 | 0.5322 | 0.5321 | 0.5324 | 0.5323 |
| 18 | rs8096960 | 0.5322 | 0.519 | 0.5462 | 0.5347 |
| 1 | rs6669186 | 0.5322 | 0.519 | 0.5462 | 0.5347 |
| 9 | rs10820743 | 0.5322 | 0.5408 | 0.5239 | 0.531 |
| 13 | rs2863263 | 0.5322 | 0.5408 | 0.5239 | 0.531 |
| 9 | rs10810541 | 0.5322 | 0.5918 | 0.4835 | 0.5292 |
| 2 | rs1357378 | 0.5322 | 0.516 | 0.5494 | 0.5353 |
| 12 | rs10774832 | 0.5322 | 0.516 | 0.5494 | 0.5353 |
| 13 | rs12853508 | 0.5322 | 0.5087 | 0.5579 | 0.5372 |
| 13 | rs1865351 | 0.5322 | 0.5044 | 0.5632 | 0.5384 |
| 3 | rs17640527 | 0.5322 | 0.5044 | 0.5632 | 0.5384 |
| 1 | rs4420153 | 0.5322 | 0.5525 | 0.5133 | 0.5298 |
| 6 | rs9296163 | 0.5322 | 0.5525 | 0.5133 | 0.5298 |
| 14 | rs12147332 | 0.5322 | 0.5525 | 0.5133 | 0.5298 |
| 17 | rs17808354 | 0.5321 | 0.5131 | 0.5526 | 0.536 |
| 4 | rs2604560 | 0.5321 | 0.5131 | 0.5526 | 0.536 |
| 9 | rs11103793 | 0.5321 | 0.5131 | 0.5526 | 0.536 |
| 8 | rs4737240 | 0.5321 | 0.5452 | 0.5197 | 0.5304 |
| 4 | rs962488 | 0.5321 | 0.5452 | 0.5197 | 0.5304 |
| 9 | rs10982572 | 0.5321 | 0.4869 | 0.5866 | 0.5446 |
| 21 | rs2830041 | 0.5321 | 0.5612 | 0.5058 | 0.5292 |
| 22 | rs5755582 | 0.5321 | 0.5612 | 0.5058 | 0.5292 |
| 5 | rs13182989 | 0.5321 | 0.484 | 0.5909 | 0.5458 |
| 16 | rs2875710 | 0.5321 | 0.4738 | 0.6068 | 0.5507 |
| 8 | rs2164548 | 0.5321 | 0.535 | 0.5292 | 0.5317 |
| 9 | rs4629949 | 0.5321 | 0.535 | 0.5292 | 0.5317 |
| 20 | rs6109675 | 0.5321 | 0.535 | 0.5292 | 0.5317 |
| 6 | rs3131630 | 0.5321 | 0.6327 | 0.4591 | 0.5323 |
| 4 | rs10518331 | 0.5321 | 0.5394 | 0.525 | 0.531 |
| 7 | rs13238446 | 0.5321 | 0.5394 | 0.525 | 0.531 |
| 6 | rs7761904 | 0.5321 | 0.551 | 0.5143 | 0.5298 |
| 17 | rs9904457 | 0.532 | 0.5306 | 0.5335 | 0.5323 |
| 7 | rs17663622 | 0.532 | 0.5306 | 0.5335 | 0.5323 |
| 11 | rs7925701 | 0.532 | 0.5962 | 0.4803 | 0.5292 |
| 1 | rs506778 | 0.532 | 0.4811 | 0.5951 | 0.547 |
| 9 | rs4741761 | 0.532 | 0.5058 | 0.5611 | 0.5378 |
| 2 | rs871274 | 0.532 | 0.5598 | 0.5069 | 0.5292 |
| 22 | rs873387 | 0.532 | 0.5598 | 0.5069 | 0.5292 |
| 1 | rs12066114 | 0.532 | 0.4927 | 0.5781 | 0.5421 |
| 1 | rs12066114 | 0.532 | 0.4927 | 0.5781 | 0.5421 |
| 9 | rs7041664 | 0.532 | 0.5204 | 0.5441 | 0.5341 |
| 6 | rs17750385 | 0.532 | 0.5437 | 0.5207 | 0.5304 |
| 2 | rs6434040 | 0.532 | 0.5175 | 0.5473 | 0.5347 |
| 3 | rs4678136 | 0.532 | 0.5175 | 0.5473 | 0.5347 |
| 1 | rs6668917 | 0.532 | 0.5175 | 0.5473 | 0.5347 |
| X | rs4612534 | 0.532 | 0.5015 | 0.5664 | 0.539 |
| 4 | rs13140329 | 0.532 | 0.5015 | 0.5664 | 0.539 |
| 3 | rs9835425 | 0.532 | 0.5015 | 0.5664 | 0.539 |
| 5 | rs156322 | 0.5319 | 0.5496 | 0.5154 | 0.5298 |
| 1 | rs4845812 | 0.5319 | 0.5496 | 0.5154 | 0.5298 |
| 1 | rs4845812 | 0.5319 | 0.5496 | 0.5154 | 0.5298 |
| 19 | rs4803260 | 0.5319 | 0.5496 | 0.5154 | 0.5298 |
| 7 | rs41321 | 0.5319 | 0.5146 | 0.5505 | 0.5353 |
| 12 | rs4503583 | 0.5319 | 0.5146 | 0.5505 | 0.5353 |
| 1 | rs4658435 | 0.5319 | 0.5379 | 0.526 | 0.531 |
| 3 | rs6767543 | 0.5319 | 0.5379 | 0.526 | 0.531 |
| 10 | rs11003158 | 0.5319 | 0.5335 | 0.5303 | 0.5317 |
| 14 | rs7144510 | 0.5319 | 0.5335 | 0.5303 | 0.5317 |
| 17 | rs11079671 | 0.5319 | 0.5073 | 0.559 | 0.5372 |
| 2 | rs10496575 | 0.5319 | 0.5569 | 0.509 | 0.5292 |
| 5 | rs2169519 | 0.5319 | 0.5569 | 0.509 | 0.5292 |
| 1 | rs500586 | 0.5319 | 0.5773 | 0.4931 | 0.5286 |
| 5 | rs248342 | 0.5319 | 0.5773 | 0.4931 | 0.5286 |
| 6 | rs3095254 | 0.5319 | 0.5787 | 0.492 | 0.5286 |
| 13 | rs1571316 | 0.5319 | 0.5758 | 0.4942 | 0.5286 |
| 11 | rs580301 | 0.5319 | 0.5758 | 0.4942 | 0.5286 |
| 17 | rs312742 | 0.5319 | 0.6166 | 0.4676 | 0.5304 |
| X | rs5991106 | 0.5319 | 0.5743 | 0.4952 | 0.5286 |
| 18 | rs7230462 | 0.5318 | 0.6093 | 0.4718 | 0.5298 |
| 19 | rs8103441 | 0.5318 | 0.5816 | 0.4899 | 0.5286 |
| 16 | rs8053890 | 0.5318 | 0.5029 | 0.5643 | 0.5384 |
| 17 | rs11652442 | 0.5318 | 0.5729 | 0.4963 | 0.5286 |
| 7 | rs10953704 | 0.5318 | 0.5029 | 0.5643 | 0.5384 |
| 1 | rs1575071 | 0.5318 | 0.5729 | 0.4963 | 0.5286 |
| 21 | rs220253 | 0.5318 | 0.5117 | 0.5537 | 0.536 |
| 7 | rs12154905 | 0.5318 | 0.5117 | 0.5537 | 0.536 |
| 4 | rs1480321 | 0.5318 | 0.5117 | 0.5537 | 0.536 |
| 1 | rs4428852 | 0.5318 | 0.4665 | 0.6185 | 0.5544 |
| 1 | rs4428852 | 0.5318 | 0.4665 | 0.6185 | 0.5544 |
| 10 | rs6588750 | 0.5318 | 0.5423 | 0.5218 | 0.5304 |
| 20 | rs6028687 | 0.5318 | 0.5423 | 0.5218 | 0.5304 |
| 5 | rs10045427 | 0.5318 | 0.5423 | 0.5218 | 0.5304 |
| 14 | rs11157158 | 0.5318 | 0.5481 | 0.5165 | 0.5298 |
| 13 | rs362 | 0.5318 | 0.5481 | 0.5165 | 0.5298 |
| 4 | rs2646077 | 0.5318 | 0.5481 | 0.5165 | 0.5298 |
| 4 | rs6448715 | 0.5318 | 0.5481 | 0.5165 | 0.5298 |
| 1 | rs4662154 | 0.5318 | 0.4971 | 0.5717 | 0.5403 |
| 1 | rs4970503 | 0.5318 | 0.4971 | 0.5717 | 0.5403 |
| 1 | rs4970503 | 0.5318 | 0.4971 | 0.5717 | 0.5403 |
| 10 | rs11189513 | 0.5318 | 0.4971 | 0.5717 | 0.5403 |
| 1 | rs10797493 | 0.5318 | 0.57 | 0.4984 | 0.5286 |
| 1 | rs10912915 | 0.5318 | 0.5554 | 0.5101 | 0.5292 |
| 8 | rs10958357 | 0.5318 | 0.586 | 0.4867 | 0.5286 |
| 1 | rs10802826 | 0.5318 | 0.586 | 0.4867 | 0.5286 |
| 1 | rs7518371 | 0.5318 | 0.5685 | 0.4995 | 0.5286 |
| 2 | rs7594476 | 0.5317 | 0.519 | 0.5452 | 0.5341 |
| 18 | rs11564381 | 0.5317 | 0.5219 | 0.542 | 0.5335 |
| 5 | rs13153275 | 0.5317 | 0.5087 | 0.5569 | 0.5366 |
| 13 | rs7988219 | 0.5317 | 0.5087 | 0.5569 | 0.5366 |
| 16 | rs7187172 | 0.5317 | 0.5044 | 0.5622 | 0.5378 |
| 16 | rs2292322 | 0.5317 | 0.516 | 0.5484 | 0.5347 |
| X | rs5963230 | 0.5317 | 0.5466 | 0.5175 | 0.5298 |
| 8 | rs2088349 | 0.5317 | 0.5248 | 0.5388 | 0.5329 |
| 2 | rs12464454 | 0.5317 | 0.5248 | 0.5388 | 0.5329 |
| 12 | rs11180402 | 0.5317 | 0.5248 | 0.5388 | 0.5329 |
| 14 | rs11159695 | 0.5317 | 0.4738 | 0.6057 | 0.5501 |
| 14 | rs7142759 | 0.5317 | 0.5408 | 0.5228 | 0.5304 |
| 6 | rs2253981 | 0.5317 | 0.4854 | 0.5877 | 0.5446 |
| 5 | rs4244437 | 0.5316 | 0.4825 | 0.5919 | 0.5458 |
| 3 | rs4679557 | 0.5316 | 0.4825 | 0.5919 | 0.5458 |
| 7 | rs10259665 | 0.5316 | 0.5131 | 0.5515 | 0.5353 |
| 12 | rs1708188 | 0.5316 | 0.5131 | 0.5515 | 0.5353 |
| 7 | rs6976173 | 0.5316 | 0.5131 | 0.5515 | 0.5353 |
| 6 | rs9367137 | 0.5316 | 0.5277 | 0.5356 | 0.5323 |
| 3 | rs6780103 | 0.5316 | 0.5277 | 0.5356 | 0.5323 |
| 5 | rs1862136 | 0.5316 | 0.4913 | 0.5792 | 0.5421 |
| 3 | rs4563435 | 0.5316 | 0.6122 | 0.4697 | 0.5298 |
| 19 | rs11668315 | 0.5316 | 0.6122 | 0.4697 | 0.5298 |
| 9 | rs12348328 | 0.5316 | 0.5525 | 0.5122 | 0.5292 |
| 22 | rs6005221 | 0.5316 | 0.5627 | 0.5037 | 0.5286 |
| 22 | rs1543416 | 0.5316 | 0.5627 | 0.5037 | 0.5286 |
| 2 | rs1078819 | 0.5316 | 0.5452 | 0.5186 | 0.5298 |
| 16 | rs7404896 | 0.5316 | 0.5058 | 0.56 | 0.5372 |
| 6 | rs9266636 | 0.5316 | 0.4927 | 0.577 | 0.5415 |
| 8 | rs11992536 | 0.5316 | 0.535 | 0.5282 | 0.531 |
| 6 | rs1080085 | 0.5315 | 0.5102 | 0.5547 | 0.536 |
| 8 | rs10503896 | 0.5315 | 0.5394 | 0.5239 | 0.5304 |
| 4 | rs1541374 | 0.5315 | 0.5394 | 0.5239 | 0.5304 |
| 2 | rs6547521 | 0.5315 | 0.5306 | 0.5324 | 0.5317 |
| 2 | rs4853630 | 0.5315 | 0.5306 | 0.5324 | 0.5317 |
| 17 | rs12601852 | 0.5315 | 0.4781 | 0.5983 | 0.5476 |
| 6 | rs3094006 | 0.5315 | 0.4781 | 0.5983 | 0.5476 |
| 8 | rs6472849 | 0.5315 | 0.5612 | 0.5048 | 0.5286 |
| 6 | rs3793017 | 0.5315 | 0.5612 | 0.5048 | 0.5286 |
| 10 | rs4570530 | 0.5315 | 0.5612 | 0.5048 | 0.5286 |
| 7 | rs10278217 | 0.5315 | 0.4942 | 0.5749 | 0.5409 |
| 2 | rs1516920 | 0.5315 | 0.5204 | 0.543 | 0.5335 |
| 17 | rs963987 | 0.5315 | 0.5204 | 0.543 | 0.5335 |
| 6 | rs1321354 | 0.5315 | 0.5204 | 0.543 | 0.5335 |
| 10 | rs4918575 | 0.5315 | 0.5175 | 0.5462 | 0.5341 |
| 15 | rs4533267 | 0.5315 | 0.5175 | 0.5462 | 0.5341 |
| 11 | rs11218533 | 0.5315 | 0.5175 | 0.5462 | 0.5341 |
| 8 | rs3739262 | 0.5315 | 0.5175 | 0.5462 | 0.5341 |
| 2 | rs1825374 | 0.5315 | 0.4708 | 0.61 | 0.5513 |
| 11 | rs3781578 | 0.5315 | 0.5948 | 0.4803 | 0.5286 |
| X | rs806641 | 0.5314 | 0.5598 | 0.5058 | 0.5286 |
| 4 | rs11939334 | 0.5314 | 0.5598 | 0.5058 | 0.5286 |
| 10 | rs10905106 | 0.5314 | 0.5598 | 0.5058 | 0.5286 |
| 12 | rs621830 | 0.5314 | 0.5146 | 0.5494 | 0.5347 |
| 5 | rs2366774 | 0.5314 | 0.4956 | 0.5728 | 0.5403 |
| 6 | rs2170185 | 0.5314 | 0.5437 | 0.5197 | 0.5298 |
| 10 | rs1555961 | 0.5314 | 0.5437 | 0.5197 | 0.5298 |
| 3 | rs4305453 | 0.5314 | 0.5437 | 0.5197 | 0.5298 |
| 10 | rs4074771 | 0.5314 | 0.5262 | 0.5367 | 0.5323 |
| 6 | rs9392436 | 0.5314 | 0.5262 | 0.5367 | 0.5323 |
| 22 | rs4820414 | 0.5314 | 0.5962 | 0.4793 | 0.5286 |
| 9 | rs10817517 | 0.5314 | 0.5496 | 0.5143 | 0.5292 |
| 9 | rs7044017 | 0.5314 | 0.5496 | 0.5143 | 0.5292 |
| 4 | rs9307203 | 0.5314 | 0.5583 | 0.5069 | 0.5286 |
| 8 | rs2688325 | 0.5314 | 0.5335 | 0.5292 | 0.531 |
| 8 | rs2589750 | 0.5314 | 0.5335 | 0.5292 | 0.531 |
| 10 | rs11255917 | 0.5314 | 0.5335 | 0.5292 | 0.531 |
| 8 | rs452375 | 0.5314 | 0.5029 | 0.5632 | 0.5378 |
| 11 | rs4375421 | 0.5314 | 0.5335 | 0.5292 | 0.531 |
| 6 | rs11153277 | 0.5313 | 0.5117 | 0.5526 | 0.5353 |
| 21 | rs2831482 | 0.5313 | 0.5117 | 0.5526 | 0.5353 |
| 18 | rs789030 | 0.5313 | 0.4971 | 0.5707 | 0.5396 |
| 21 | rs11702354 | 0.5313 | 0.4971 | 0.5707 | 0.5396 |
| 2 | rs12612384 | 0.5313 | 0.6152 | 0.4676 | 0.5298 |
| 6 | rs6926162 | 0.5313 | 0.5292 | 0.5335 | 0.5317 |
| 17 | rs11652288 | 0.5313 | 0.5292 | 0.5335 | 0.5317 |
| 7 | rs7785513 | 0.5313 | 0.5292 | 0.5335 | 0.5317 |
| 21 | rs1467385 | 0.5313 | 0.5292 | 0.5335 | 0.5317 |
| 11 | rs948461 | 0.5313 | 0.5292 | 0.5335 | 0.5317 |
| 1 | rs17433222 | 0.5313 | 0.5569 | 0.508 | 0.5286 |
| 1 | rs17433222 | 0.5313 | 0.5569 | 0.508 | 0.5286 |
| 13 | rs4462469 | 0.5313 | 0.5569 | 0.508 | 0.5286 |
| 6 | rs9349280 | 0.5313 | 0.5569 | 0.508 | 0.5286 |
| 19 | rs3752193 | 0.5313 | 0.6079 | 0.4718 | 0.5292 |
| 6 | rs1264704 | 0.5312 | 0.4985 | 0.5685 | 0.539 |
| 9 | rs1449653 | 0.5312 | 0.4985 | 0.5685 | 0.539 |
| 12 | rs7973936 | 0.5312 | 0.5758 | 0.4931 | 0.528 |
| 2 | rs10164964 | 0.5312 | 0.5743 | 0.4942 | 0.528 |
| 3 | rs6788569 | 0.5312 | 0.5802 | 0.4899 | 0.528 |
| 15 | rs16941421 | 0.5312 | 0.5044 | 0.5611 | 0.5372 |
| 4 | rs7670248 | 0.5312 | 0.519 | 0.5441 | 0.5335 |
| 8 | rs10956453 | 0.5312 | 0.5087 | 0.5558 | 0.536 |
| 6 | rs6907985 | 0.5312 | 0.5087 | 0.5558 | 0.536 |
| 1 | rs1332636 | 0.5312 | 0.5087 | 0.5558 | 0.536 |
| 6 | rs12202204 | 0.5312 | 0.5219 | 0.5409 | 0.5329 |
| 7 | rs272665 | 0.5312 | 0.5219 | 0.5409 | 0.5329 |
| 14 | rs5008080 | 0.5312 | 0.4825 | 0.5909 | 0.5452 |
| 13 | rs7324651 | 0.5312 | 0.5714 | 0.4963 | 0.528 |
| 3 | rs12497287 | 0.5312 | 0.5554 | 0.509 | 0.5286 |
| 3 | rs6778968 | 0.5312 | 0.516 | 0.5473 | 0.5341 |
| 11 | rs7949201 | 0.5312 | 0.4898 | 0.5802 | 0.5421 |
| X | rs12012053 | 0.5312 | 0.5364 | 0.526 | 0.5304 |
| 4 | rs4572833 | 0.5312 | 0.57 | 0.4973 | 0.528 |
| 3 | rs3846051 | 0.5312 | 0.5321 | 0.5303 | 0.531 |
| 1 | rs2799567 | 0.5312 | 0.5248 | 0.5377 | 0.5323 |
| 11 | rs639187 | 0.5312 | 0.5248 | 0.5377 | 0.5323 |
| 3 | rs11923660 | 0.5312 | 0.5845 | 0.4867 | 0.528 |
| 2 | rs1521756 | 0.5311 | 0.5131 | 0.5505 | 0.5347 |
| 18 | rs4799930 | 0.5311 | 0.5131 | 0.5505 | 0.5347 |
| 8 | rs1054748 | 0.5311 | 0.4796 | 0.5951 | 0.5464 |
| 16 | rs17193922 | 0.5311 | 0.5466 | 0.5165 | 0.5292 |
| 3 | rs4688077 | 0.5311 | 0.5466 | 0.5165 | 0.5292 |
| 7 | rs10246131 | 0.5311 | 0.5408 | 0.5218 | 0.5298 |
| 2 | rs2570504 | 0.5311 | 0.5408 | 0.5218 | 0.5298 |
| 3 | rs489683 | 0.5311 | 0.5408 | 0.5218 | 0.5298 |
| 16 | rs8063904 | 0.5311 | 0.5539 | 0.5101 | 0.5286 |
| 16 | rs9926829 | 0.5311 | 0.5539 | 0.5101 | 0.5286 |
| 2 | rs13425179 | 0.5311 | 0.5539 | 0.5101 | 0.5286 |
| 6 | rs2532925 | 0.5311 | 0.4927 | 0.576 | 0.5409 |
| 1 | rs10752775 | 0.5311 | 0.4927 | 0.576 | 0.5409 |
| 4 | rs4689260 | 0.5311 | 0.5277 | 0.5345 | 0.5317 |
| 13 | rs2181751 | 0.5311 | 0.5277 | 0.5345 | 0.5317 |
| 11 | rs7102878 | 0.5311 | 0.5277 | 0.5345 | 0.5317 |
| X | rs498207 | 0.5311 | 0.5875 | 0.4846 | 0.528 |
| 2 | rs231390 | 0.5311 | 0.4781 | 0.5972 | 0.547 |
| 3 | rs6803442 | 0.5311 | 0.4781 | 0.5972 | 0.547 |
| 3 | rs2242316 | 0.5311 | 0.5058 | 0.559 | 0.5366 |
| 2 | rs7601793 | 0.5311 | 0.5058 | 0.559 | 0.5366 |
| 15 | rs1579821 | 0.5311 | 0.5058 | 0.559 | 0.5366 |
| 3 | rs9818164 | 0.5311 | 0.5058 | 0.559 | 0.5366 |
| 4 | rs2995917 | 0.5311 | 0.4708 | 0.6089 | 0.5507 |
| 3 | rs4687100 | 0.531 | 0.5102 | 0.5537 | 0.5353 |
| 11 | rs4121977 | 0.531 | 0.5102 | 0.5537 | 0.5353 |
| 3 | rs7644703 | 0.531 | 0.602 | 0.475 | 0.5286 |
| 12 | rs3782578 | 0.531 | 0.5641 | 0.5016 | 0.528 |
| 12 | rs10862407 | 0.531 | 0.5641 | 0.5016 | 0.528 |

Table S2. Prediction accuracy with Bootstrap mean and 95% confidence interval for optimal SNP subsets using LDA or sIB for predicting psoriasis

| Subsets | Components  (dbSNP_rs  on chromosome) | CV  HMSS | CV accuracy among controls | CV  accuracy among cases | Total CV accuracy | Test HMSS | Test accuracy among controls | Test accuracy among cases | Total  test  accuracy |
| --- | --- | --- | --- | --- | --- | --- | --- | --- | --- |
| **LDA** |  |  |  |  |  |  |  |  |  |
| 1 SNP* | rs10905106 on 10 | 0.498(0.498, 0.475-0.518) | 0.520(0.518, 0.482-0.551) | 0.477(0.480, 0.449-0.508) | 0.495(0.496, 0.474-0.516) | 0.544(0.494, 0.469-0.519) | 0.574(0.540, 0.510-0.572) | 0.517(0.457,  0.417-0.496) | 0.553(0.508, 0.486-0.532) |
| 2 SNPs* | rs10958357 on 8  rs7973936 on 12 | 0.486(0.499, 0.480-0.520) | 0.450(0.500, 0.450-0.542) | 0.527(0.501, 0.461-0.548) | 0.495(0.500, 0.481-0.521) | 0.556(0.498, 0.471-0.525) | 0.588(0.545, 0.521-0.579) | 0.528(0.459, 0.420-0.499) | 0.565(0.512, 0.491-0.535) |
| 1 SNP^∆^ | rs4375421 on  11 | 0.540(0.497, 0.474-0.519) | 0.544(0.496, 0.459-0.531) | 0.537(0.499, 0.473-0.524) | 0.540(0.498, 0.476-0.518) | 0.492(0.500, 0.476-0.529) | 0.488(0.494, 0.466-0.528) | 0.497(0.507,  0.468-0.550) | 0.491(0.499, 0.474-0.529) |
| 2 SNPs^∆^ | rs950753 on 3  rs7058025 on X | 0.570(0.493, 0.468-0.514) | 0.548(0.453, 0.388-0.541) | 0.594(0.547, 0.466-0.613) | 0.575(0.508, 0.486-0.530) | 0.463(0.476, 0.451-0.502) | 0.383(0.395, 0.367-0.425) | 0.585(0.601, 0.562-0.636) | 0.459(0.473, 0.450-0.498) |
| FS | 38 SNPs | 0.604(0.500, 0.478-0.520) | 0.621(0.483, 0.456-0.516) | 0.591(0.518, 0.494-0.542) | 0.622(0.503, 0.482-0.523) | **0.520(0.496, 0.472-0.524)** | **0.488(0.467, 0.436-0.499)** | **0.558(0.529, 0.494-0.569)** | **0.514(0.491, 0.466-0.518)** |
| SFFS | 32 SNPs | 0.622(0.497, 0.475-0.520) | 0.622(0.474, 0.441-0.504) | 0.622(0.523, 0.496-0.547) | 0.622(0.502, 0.479-0.525) | 0.512(0.498, 0.472-0.523) | 0.5 00(0.497, 0.467-0.534) | 0.524(0.500, 0.462-0.546) | 0.509(0.498, 0.473-0.522) |
| **sIB** |  |  |  |  |  |  |  |  |  |
| 1 SNP* | rs12191877 on 6 | 0.611(0.605, 0.563-0.630) | 0.722(0.723, 0.693-0.762) | 0.530(0.523, 0.459-0.555) | 0.611(0.608, 0.580-0.631) | 0.668(0.668, 0.641-0.694) | 0.761(0.760, 0.735-0.785) | 0.596(0.596, 0.560-0.632) | 0.699(0.698, 0.676-0.720) |
| 2 SNPs* | rs12191877 on 6  rs4953658 on 2 | 0.557(0.444, 0.014-0.633) | 0.757(0.800, 0.657-1.00) | 0.441(0.367, 0.007-0.596) | 0.574(0.550, 0.426-0.633) | **0.674(0.674, 0.650-0.698)** | **0.712(0.710, 0.684-0.737)** | **0.641(0.642, 0.604-0.676)** | **0.685(0.684, 0.662-0.707)** |
| FS | rs12191877 on 6 | 0.611(0.605, 0.563-0.630) | 0.722(0.723, 0.693-0.762) | 0.530(0.523, 0.459-0.555) | 0.611(0.608, 0.580-0.631) | 0.668(0.668, 0.641-0.694) | 0.761(0.760, 0.735-0.785) | 0.596(0.596, 0.560-0.632) | 0.699(0.698, 0.676-0.720) |
| SFFS | rs2844627 on 6  rs7773175 on 6 | 0.619(0.617, 0.576-0.641) | 0.690(0.693, 0.655-0.735) | 0.562(0.558, 0.476-0.595) | 0.616(0.615, 0.585-0.638) | 0.659(0.658, 0.633-0.683) | 0.718(0.717, 0.690-0.743) | 0.609(0.610, 0.572-0.644) | 0.677(0.676, 0.655-0.699) |

* The best test HMSS among all subsets

∆ Test HMSS for the subset with the best CV HMSS

Table S3. Classification accuracy(Bootstrap mean and 95% CI) and chi-square test for 20 SNPs with the highest training HMSS by LDA for predicting psoriasis

| SNP_RS | Chr | LDA | | | | sIB | | | | *P*-value(GRU) * | *P*-value(ADO) * |
| --- | --- | --- | --- | --- | --- | --- | --- | --- | --- | --- | --- |
|  |  | Training HMSS | Training  Accuracy | Test HMSS | Test  Accuracy | Training HMSS | Training  Accuracy | Test HMSS | Test  Accuracy |  |  |
| rs12191877 | 6 | 0.611(0.611,  0.590-0.630) | 0.611(0.611,  0.590-0.631) | 0.315(0.468,  0.435-0.502) | 0.417(0.531,  0.507-0.556) | 0.611(0.606,  0.588-0.630) | 0.611(0.609,  0.588-0.631) | 0.668(0.668,  0.641-0.694) | 0.699(0.698,  0.676-0.720) | **0** | **0** |
| rs2894207 | 6 | 0.603(0.603,  0.583-0.622) | 0.601(0.600,  0.580-0.619) | 0.387(0.494,  0.464-0.522) | 0.420(0.512,  0.488-0.537) | 0.603(0.585,  0.573-0.622) | 0.601(0.595,  0.570-0.619) | 0.657(0.656,  0.636-0.680) | 0.661(0.660,  0.638-0.684) | **0** | **1.11×10^-16^** |
| rs3130517 | 6 | 0.600(0.599,  0.575-0.618) | 0.613(0.612,  0.592-0.629) | 0.414(0.490,  0.465-0.516) | 0.408(0.484,  0.458-0.511) | 0.600(0.599,  0.575-0.618) | 0.613(0.612,  0.592-0.629) | 0.608(0.609,  0.583-0.636) | 0.600(0.601,  0.575-0.630) | **0** | **4.33×10^-15^** |
| rs2394895 | 6 | 0.598(0.597,  0.577-0.615) | 0.599(0.597,  0.578-0.616) | 0.425(0.500,  0.475-0.530) | 0.424(0.496,  0.472-0.526) | 0.598(0.595,  0.576-0.615) | 0.599(0.597,  0.577-0.615) | 0.620(0.620,  0.595-0.651) | 0.611(0.612,  0.585-0.643) | **0** | **1.13×10^-14^** |
| rs2844627 | 6 | 0.598(0.596,  0.574-0.616) | 0.613(0.612,  0.591-0.630) | 0.413(0.496,  0.473-0.524) | 0.409(0.490,  0.467-0.518) | 0.598(0.564,  0.269-0.616) | 0.613(0.600,  0.486-0.630) | 0.627(0.626,  0.604-0.653) | 0.617(0.617,  0.593-0.643) | **0** | **0** |
| rs3130713 | 6 | 0.597(0.595,  0.574-0.615) | 0.609(0.607,  0.588-0.625) | 0.400(0.488,  0.463-0.515) | 0.394(0.482,  0.457-0.509) | 0.597(0.594,  0.573-0.615) | 0.695(0.607,  0.588-0.625) | 0.599(0.600,  0.574-0.627) | 0.592(0.592,  0.567-0.619) | **5.55×10^-16^** | **1.52×10^-14^** |
| rs3130467 | 6 | 0.596(0.595,  0.572-0.614) | 0.612(0.610,  0.590-0.628) | 0.415(0.489,  0.465-0.515) | 0.409(0.482,  0.458-0.508) | 0.596(0.595,  0.572-0.614) | 0.612(0.610,  0.590-0.628) | 0.605(0.606,  0.579-0.633) | 0.597(0.598,  0.572-0.628) | **1.11×10^-16^** | **2.73×10^-14^** |
| rs9468933 | 6 | 0.595(0.595,  0.572-0.615) | 0.597(0.597,  0.578-0.619) | 0.321(0.467,  0.435-0.496) | 0.422(0.531,  0.507-0.555) | 0.595(0.595,  0.572-0.615) | 0.597(0.597,  0.578-0.619) | 0.656(0.655,  0.627-0.681) | 0.688(0.688,  0.666-0.711) | **0** | **0** |
| rs7773175 | 6 | 0.585(0.584,0.560-0.606) | 0.610(0.610,  0.589-0.631) | 0.416(0.486,  0.463-0.508) | 0.410(0.480,  0.456-0.501) | 0.585(0.578,  0.556-0.606) | 0.610(0.607,  0.584-0.631) | 0.604(0.601,  0.577-0.627) | 0.596(0.594,  0.571-0.619) | **0** | **1.33×10^-15^** |
| rs6861600 | 5 | 0.569(0.569,  0.549-0.591) | 0.569(0.569,  0.549-0.589) | 0.456(0.497,  0.521-0.475) | 0.452(0.492,  0.514-0.470) | 0.569(0.530,  0.201-0.591) | 0.569(0.573,  0.551-0.595) | 0.544(0.544,  0.521-0.568) | 0.537(0.537,  0.513-0.560) | 7.48×10^-4^ | **2.32×10^-8^** |
| rs9380237 | 6 | 0.569(0.567,  0.545-0.588) | 0.593(0.592,  0.571-0.613) | 0.405(0.492,  0.468-0.522) | 0.400(0.485,  0.462-0.515) | 0.569(0.540,  0.258-0.588) | 0.593(0.582,  0.477-0.613) | 0.628(0.627,  0.604-0.651) | 0.618(0.618,  0.595-0.643) | **0** | **1.38×10^-10^** |
| rs6887695 | 5 | 0.568(0.568,  0.546-0.589) | 0.568(0.568,  0.548-0.588) | 0.454(0.498,  0.475-0.521) | 0.450(0.492,  0.470-0.514) | 0.568(0.528,  0.198-0.589) | 0.568(0.572,  0.550-0.595) | 0.546(0.546,  0.523-0.568) | 0.539(0.539,  0.515-0.563) | 7.46 ×10^-4^ | **3.96×10^-8^** |
| rs3823418 | 6 | 0.568(0.567,  0.545-0.588) | 0.574(0.574,  0.552-0.593) | 0.342(0.465,  0.430-0.500) | 0.441(0.533,  0.508-0.559) | 0.120(0.335,  0.103-0.587) | 0.451(0.513,  0.437-0.593) | 0.142(0.144,  0.105-0.177) | 0.637(0.637,  0.616-0.661) | **0** | **1.11×10^-16^** |
| rs1265078 | 6 | 0.565(0.565,  0.545-0.589) | 0.599(0.599,  0.579-0.618) | 0.434(0.477,  0.453-0.504) | 0.431(0.474,  0.454-0.499) | 0.565(0.516,  0.267-0.588) | 0.599(0.581,  0.481-0.618) | 0.561(0.560,  0.536-0.587) | 0.558(0.568,  0.532-0.583) | **5.29×10^-11^** | **3.44×10^-12^** |
| rs2647087 | 6 | 0.564(0.563,  0.543-0.582) | 0.574(0.573,  0.553-0.591) | 0.443(0.498,  0.473-0.524) | 0.446(0.498,  0.472-0.524) | 0.564(0.495,  0.239-0.582) | 0.574(0.553,  0.467-0.591) | 0.573(0.574,  0.548-0.601) | 0.569(0.569,  0.544-0.596) | **1.25×10^-8^** | **1.21×10^-8^** |
| rs2858333 | 6 | 0.564(0.563,  0.543-0.582) | 0.573(0.572,  0.553-0.590) | 0.444(0.498,  0.473-0.525) | 0.446(0.497,  0.471-0.523) | 0.564(0.477,  0.242-0.582) | 0.573(0.546,  0.464-0.590) | 0.572(0.572,  0.547-0.599) | 0.567(0.567,  0.543-0.594) | **5.65×10^-8^** | **1.23×10^-8^** |
| rs3132965 | 6 | 0.564(0.564,  0.542-0.586) | 0.561(0.562,  0.540-0.582) | 0.434(0.490,  0.460-0.520) | 0.471(0.518,  0.494-0.542) | 0.564(0.376,  0.107-0.584) | 0.561(0.516,  0.437-0.581) | 0.574(0.575,  0.548-0.604) | 0.592(0.593,  0.568-0.618) | **2.57×10^-9^** | **6.62×10^-9^** |
| rs10947208 | 6 | 0.563(0.563,  0.541-0.583) | 0.560(0.561,  0.539-0.580) | 0.442(0.496,  0.471-0.520) | 0.455(0.514,  0.481-0.527) | 0.563(0.424,  0.183-0.581) | 0.560(0.526,  0.451-0.578) | 0.554(0.554,  0.530-0.578) | 0.558(0.558,  0.535-0.579) | 2.77×10^-5^ | **3.71×10^-8^** |
| rs9266846 | 6 | 0.562(0.562,  0.544-0.582) | 0.562(0.563,  0.544-0.583) | 0.458(0.499,  0.474-0.525) | 0.462(0.500,  0.476-0.524) | 0.562(0.434,  0.204-0.580) | 0.562(0.529,  0.454-0.582) | 0.548(0.548,  0.524-0.571) | 0.546(0.546,  0.523-0.569) | 1.31×10^-5^ | 3.03×10^-7^ |
| rs497150 | 22 | 0.562(0.561,  0.541-0.581) | 0.558(0.558,  0.538-0.578) | 0.486(0.493,  0.470-0.520) | 0.507(0.514,  0.492-0.525) | 0.562(0.554,  0.526-0.581) | 0.558(0.555,  0.532-0.578) | 0.490(0.488,  0.462-0.517) | 0.511(0.510,  0.487-0.536) | 0.22062 | 1.67×10^-5^ |

* cut-off *P*-value=1.11×10^-7^ (0.05/451724)
